# A new crustacean from the Herefordshire (Silurian) Lagerstätte, UK, and its significance in malacostracan evolution

**DOI:** 10.1098/rspb.2017.0279

**Published:** 2017-03-22

**Authors:** David J. Siveter, Derek E. G. Briggs, Derek J. Siveter, Mark D. Sutton, David Legg

**Affiliations:** 1 Department of Geology, University of Leicester, Leicester LE1 7RH, UK; 2 Department of Geology and Geophysics, and Yale Peabody Museum of Natural History, Yale University, PO Box 208109, New Haven, CT 06520-8109, USA; 3 Earth Collections, University Museum of Natural History, Oxford OX1 3PW, UK; 4 Department of Earth Sciences, University of Oxford, South Parks Road, Oxford OX1 3AN, UK; 5 Department of Earth Sciences and Engineering, Imperial College London, London SW7 2BP, UK

**Keywords:** Crustacea, Herefordshire Lagerstätte, Malacostraca, Phyllocarida, Leptostraca, Silurian

## Abstract

*Cascolus ravitis* gen. et sp. nov. is a three-dimensionally preserved fossil crustacean with soft parts from the Herefordshire (Silurian) Lagerstätte, UK. It is characterized by a head with a head shield and five limb pairs, and a thorax (pereon) with nine appendage-bearing segments followed by an apodous abdomen (pleon). All the appendages except the first are biramous and have a gnathobase. The post-mandibular appendages are similar one to another, and bear petal-shaped epipods that probably functioned as a part of the respiratory–circulatory system. Cladistic analysis resolves the new taxon as a stem-group leptostracan (Malacostraca). This well-preserved arthropod provides novel insights into the evolution of appendage morphology, tagmosis and the possible respiratory–circulatory physiology of a basal malacostracan.

## 1. Introduction

Arthropods are the most diverse group of organisms in both the fossil record and the modern biota. The group originated in the Ediacaran and crown-group representatives occur in the early Cambrian [1,2]. Although arthropod phylogeny has generated huge debate, a combination of morphological and molecular data and evidence from fossils has increased consensus regarding the interrelationships of the major groups within the phylum (e.g. [3–6]). Crustaceans are the most abundant fossil arthropods, and the major pancrustacean groups first occur, or are supposedly present, in the Cambrian [2,6,7]. Here, we describe a new Silurian crustacean with preserved soft parts, *Cascolus ravitis* gen. et sp. nov., from the Herefordshire (Silurian) Lagerstätte, UK. *Cascolus* is resolved as a member of the stem-group of Leptostraca. Leptostracans are known almost entirely from living representatives. Leptostraca and the fossil archaeostracans constitute the Phyllocarida, which is normally considered sister to Eumalacostraca [4,8], and together they constitute Malacostraca. *Cascolus* provides important clues into the morphological evolution of the sister taxon of Eumalacostraca and of the Malacostraca, one of the major groups of Crustacea.

The Herefordshire Konservat-Lagerstätte in the Welsh Borderland, UK, is globally important as a source of unparalleled palaeobiological and phylogenetic data on a diversity of Mid-Silurian (approx. 430 Myr BP) invertebrate animals [9,10]. These include a brachiopod, a polychaete worm, aplacophorans, a gastropod, a stem-group asteroid, and most abundantly a range of arthropods comprising a stem-group euarthropod, a pycnogonid and other chelicerates, a marrellomorph, stem-group mandibulates, four ostracod species, a barnacle, a phyllocarid and a pentastomid [10–20]. Many additional forms, including a wide variety of sponge species, brachiopods, molluscs, arthropods and echinoderms, await study.

## 2. Material and methods

**Figure 1.**
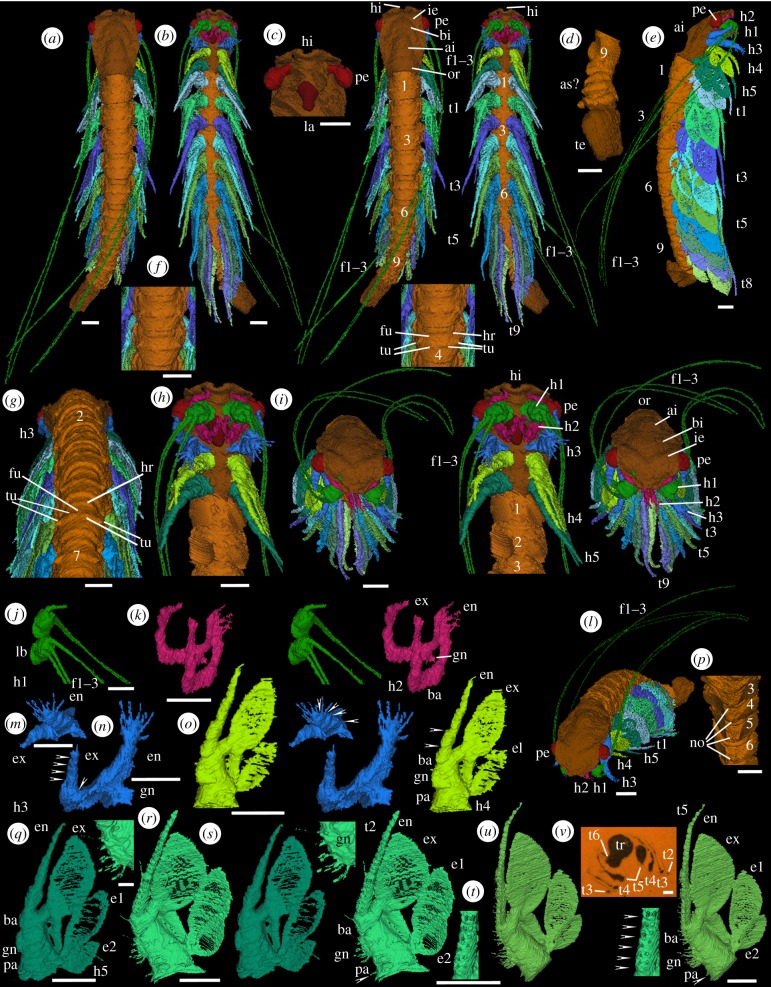
Holotype of *Cascolus ravitis,* exoskeleton and soft parts (OUMNH C.29698): (*a–u*) ‘virtual’ reconstructions (*a*,*b*,*f*,*h–k*,*m–u* are stereo-pairs); (*v*) specimen in rock. The exact boundary between structures such as body and limbs, as indicated by colour changes, is somewhat arbitrary. (*a*) Dorsal view. (*b*) Ventral view. (*c*) Anterior part of head, appendages omitted, ventral view. (*d*) Posterior part of trunk, appendages omitted, ventral view. (*e*) Right lateral view. (*f*) Part of trunk, dorsal view. (*g*) Head and most of the trunk, appendage 1 omitted, posterodorsal view. (*h*) Head with appendages and trunk segments 1 and 2 with appendages omitted; ventral view. (*i*) Anteroventral view. (*j*) Limb base and proximal part of antennules, right ventral oblique view. (*k*) Antennae, right anteroventral oblique view. (*l*) Anterolateral view. (*m,n*) Mandible, left limb: posterior oblique (*m*) and right anteroventral oblique (*n*) views. (*o*) Maxillula, left limb, posterior oblique view. (*p*) Sternites 3–6, anteroventral view, appendages omitted. (*q*) Maxilla, left limb, posterior oblique view. (*r–t*) Trunk appendage 2, left limb: gnathobase (*r*) and complete limb (*s*) posterior oblique views; and inner proximal part of endopod (*t*) posterior oblique medial view. (*u*) Trunk appendage 5, left limb, posterior oblique view. ai, axial inflation; as?, apodous segments?; ba, basipod; bi, bell-shaped inflation; e1, e2, epipods; en, endopod; ex, exopod; f1–f3, flagella; fu, furrow; hi, indentation in anterior margin of the head shield; gn, coxal gnathobase; h1–h5, head appendages; hr, half-ring of trunk tergite; ie, inflation adaxially forward of each eye (‘eye ridge’); la, labrum; lb, limb base; no, node; or, occipital ring; pa, precoxal area; pe, pedunculate eye; t1–t9, trunk appendages; te, end of the trunk, masked by extraneous matter; tr, trunk; tu, tubercle(s). Numbers refer to trunk segments and trunk tergites/sternites as appropriate. Arrows in (*m-o*, *t*) indicate podomere boundaries. Arrow in (*s*,*u*) indicates a spine/seta. Scale bars: (*a*–*q*), (*s*–*v*) are 0.5 mm; (*r*) is 0.1 mm.

The fossils of the Herefordshire Konservat-Lagerstätte occur in calcareous nodules in a volcaniclastic deposit [21]. They are preserved as calcitic in-fills in three dimensions. Using the custom SPIERS software suite the specimens are reconstructed as ‘virtual fossils’ [22,23]. After grinding of the fossil and image capture at 20 µm intervals extraneous material was removed digitally and fossil-matrix ambiguities were resolved. ‘SPIERSview’ was used to generate colour-coded three-dimensional interactive visualizations and reconstructions (figure 1), including stereo-pairs and an animation. The Oxford University Museum of Natural History (OUMNH) houses the original datasets that resulted from serial grinding.

*Cascolus ravitis* was coded into the extensive phylogenetic dataset of Legg *et al*. [4] as modified and used by Briggs *et al*. [12] (see electronic supplementary material, tables S1 and S2). This dataset, now composed of 316 taxa and 754 characters, was analysed under general parsimony in TNT v. 1.1 [24]. All characters were unordered and weighted using implied weighting with a concavity constant of three. Tree searches used 100 random addition sequences with parsimony ratchet [25], sectorial searches, tree drifting and tree fusing [26]. Nodal support was measured using symmetric resampling (each search using new technology searches with a change probability of 33%) and is reported as group supported/contradicted (GC) values. A Bayesian analysis of the dataset (electronic supplementary material, figure S2) failed to reach convergence after 15 million generations. The results displayed extensive polytomies and produced many branches inconsistent with most modern phylogenetic analyses of arthropods.

## 3. Systematic palaeontology

Phylum Arthropoda [27]

Subphylum Crustacea [28]

Class Malacostraca [29]

Subclass Phyllocarida [30]

Order Leptostraca [31]

Genus *Cascolus* gen. nov.

Type species *Cascolus ravitis* sp. nov.

Other species None.

### (a) Etymology

The new crustacean is named in honour of the naturalist and broadcaster Sir David Attenborough, who grew up on University College Leicester campus, in celebration of his 90th birthday. Latin *castrum* ‘stronghold’ and *colus* ‘dwelling in’; alluding to the Middle/Old English source for the surname ‘Attenborough, derived from *atten* ‘at the’ and *burgh* ‘a fortified place’. Latin *Ratae,* the Roman name for Leicester, *vita* ‘life’ and *commeatis* ‘a messenger’.

### (b) Diagnosis of genus (monotypic) and species

An elongate body comprising a head with a head shield, pedunculate eyes and five limb pairs; and a trunk consisting of a thorax (pereon) with nine limb-bearing segments and an apodous abdomen (pleon). The first appendage is uniramous and has three slender flagella longer than the body. All other appendages are biramous and have a gnathobase. The post-mandibular appendages are similar to one another, except that the fourth head appendage bears a single petal-shaped epipod, and the fifth head appendage and each trunk appendage bear two petal-shaped epipods.

### (c) Material

Only known from the holotype OUMNH C.29698 (figure 1*v*), a specimen with soft parts reconstructed in three dimensions (figure 1*a–u*).

### (d) Locality and horizon

Herefordshire, England, UK; Wenlock Series, Silurian.

### (e) Description

The body consists of a head bearing five pairs of appendages covered dorsally by a head shield and a thorax with nine tergites and corresponding limb-bearing segments behind which there are possibly two apodous segments extending into a region that is masked by extraneous matter (figure 1*a*,*b*,*d*,*e*). Total preserved length of the specimen is 8.9 mm; maximum width (1.3 mm) occurs at the head shield. All appendages except the first one in the head are biramous. The head shield is elongate and smooth, with maximum length axially and maximum width (approx. 70% of length) at just over half the distance from its anterior margin (figure 1*a*,*e*,*i*,*l*). The anterior margin of the head shield is inclined straight postero-laterally on both sides from a tiny axial indentation (figure 1*a–c*,*h*,*i*). In dorsal view the lateral margin is gently sinuous, curving adaxially around the eye. A gently inflated ridge traverses the head shield parallel to its anterior margin forward of the eyes (= ‘eye ridge’; figure 1*a*,*i*). A wide axial bell-shaped inflation lying between and posterior of the eyes transitions posteriorly into a dome-shaped axial inflation that widens gradually posteriorly (figure 1*a*,*i*). A broad, low occipital ring traverses the posterior one-sixth of the head shield (figure 1*a*,*i*). The posterior margin of the head shield extends slightly over the anterior margin of the trunk. Trunk tergites 1–4 are of a similar size whereas trunk tergites 5–9 are successively smaller (figure 1*a*,*e*,*g*). The widest (approximately mid-length) region of each tergite bears a transverse row of at least four tubercles arranged in pairs with each pair atop a weak ridge and consisting of one tubercle laterally and one slightly smaller tubercle dorsolaterally (figure 1*a,f,g*); other minor tubercles are also present. Anterior of the paired tubercles each tergite also has a shallow furrow posterior to a weak transverse ridge (= ‘half-ring’) (figure 1*a,f,g*). The posterior margin of each tergite is slightly higher than the succeeding tergite, which it overlaps to an unknown degree (figure 1*a,f,g*).

A small cone-shaped structure with a rounded end, presumed to be a labrum, projects posteroventrally at a point about 25% of the head length from the anterior margin (figure 1*b*,*c*,*h*). Head appendage 1 (antennule; light green, figure 1*b*,*e*,*h–j*,*l*) is uniramous and originates adaxially in front of the labrum just below the anterior margin of the head shield. The limb base is small, elongate spherical and projects more or less ventrally. Three long, fine, closely set thread-like flagella originate lateroventrally one below the other from the limb base. These extend posteriorly beyond the extremity of the trunk (only a proximal stub of the distalmost of the three flagella on the left limb is preserved). No podomeres (*sensu* Boxshall [32]) are discernible in the flagella. A pair of ovoid pedunculate eyes originate immediately below the anterior margin of the head shield just in front of the first appendage; the eyes project approximately 50% beyond the lateral margin of the shield (figure 1*a–e*,*h*,*i*,*l*).

Head appendages 2 to 5 and all trunk appendages are biramous. Head appendage 2 (antenna/second antenna; pink, figure 1*b*,*h*,*i,k*,*l*) originates just posterior of the attachment of the labrum. The inner edge of the limb base (representing at least a basipod) bears a gnathobasic endite, with at least one stout projection, adaxially directed towards the tip of the labrum. Both endopod and exopod are elongate and slightly tapered but short, not extending beyond the head shield. Both are flexed; it is difficult to resolve podomere boundaries, but the flexures suggest that several podmeres are present in each. The exopod is wider and longer than the endopod. The endopod extends ventrally and adaxially over the labrum. It is flexed in two regions, at about one-third length and near its distal end, and bears at least three fine, short spines/setae distally. The exopod projects abaxially forwards below the basipod of the first appendage and curves adaxially distally below the anterior margin of the head shield. Three flexures are discernible in the exopod, at about one-fifth and three-quarters length and near the tip. The nature of the termination is unknown.

Head appendage 3 (mandible; blue, figure 1*b*,*e*,*h*,*i*,*l–n*) originates just behind the labrum. Its limb base (representing at least a basipod) is large with a blade-like gnathobase on its inner margin projecting adaxially towards the position of the supposed atrium oris and bearing a row of stout projections (evident in the left limb). Both rami are stoutly developed and taper gradually. The endopod is wider and longer than the exopod and is flattened ab-adaxially. It consists of four successively smaller podomeres (evident on the left limb; figure 1*m*): podomeres 1 and 2 each have a single spine/seta (only the base is preserved) at mid-length on their inner margin and podomere 3 has two spines/setae at mid-length on its inner margin and at least one on its outer margin. The terminal podomere 4 bears a splay of at least four stout spines/setae. The exopod consists of five podomeres (evident on the left limb; figure 1*n*). The base of a tiny spine/seta is present on the outer margin of podomeres 3, 4 and 5, and there are two tiny spines/setae on the exopod tip.

Head appendage 4 (maxillula/first maxilla; lime green, figure 1*b*,*e*,*h*,*i*,*l*,*o*) originates just behind the mandible. The rami project posteroventrally. The limb base is large and elongate triangular in lateral view. It consists of a distal part (presumed basipod) to which the endopod and exopod are attached and a central part with a serrated blade (a gnathobase; interpreted to be coxal) on its inner margin that is directed anteroventrally towards the site of the presumed atrium oris. Between the gnathobase and the junction between the limb base and the presumed body wall/arthrodial membrane there is a precoxal area whose inner margin is of similar length to the gnathobase. The endopod is long, narrow, gently curved and tapers gradually. Articulations indicating at least the proximal podomere are evident (figure 1*o*). The exopod originates in a wide triangular-shaped connection between the outer margins of the presumed basipod and the coxa. The connection has broad, stout lateral margins flanking a much thinner central area. The main part of the exopod is petal-shaped and lamella-like with a weakly convex inner edge and a more strongly convex outer edge. Most of the exopod is very thin and impersistently preserved; its outer and inner margins are thicker. An epipod (here termed epipod 1) originates in a narrow connection on the outer proximal margin of the limb base; it is similar in form to the exopod but about half the size in area.

Head appendage 5 (maxilla/second maxilla; green, figure 1*b*,*e*,*h*,*i*,*l*,*q*) is similar in morphology to head appendage 4, including a presumed coxal gnathobase, but with an additional, smaller epipod (herein termed epipod 2) attached immediately proximal to epipod 1 on the outer edge of the limb base. The epipods and exopod are thin and edged by thicker margins. The exopod slightly overlaps epipod 1, which in turn slightly overlaps epipod 2 (e.g. figure 1*e*,*q*). Bases of tiny spines/setae are evident along the endopod.

Posterior to head appendage 4 all appendages up to and including trunk appendage 7 are each in turn slightly larger and more posteriorly directed; trunk appendages 8 and 9 are in turn slightly smaller. The morphology of the nine trunk appendages is similar to that of the posteriormost (fifth) head appendage. Trunk appendages 2 and 5 were selected for manual reconstruction in enhanced detail (figures 1*r–u* and 3): these reveal that the endopod consists of many short podomeres (approx. 20 in trunk appendage 2 and approx. 22 in trunk appendage 5; figure 1*s*,*u*), which become progressively shorter distally. Tiny slender spines/setae (at least two in proximal podomeres and one in more distal podomeres) project from the inner face of each podomere aligned in rows along the ramus (figure 1*t*(arrowed),*u*). A few spines/setae are also evident at mid-length on the outer face of the endopod of trunk appendage 2 (figure 1*s*). The gnathobase bears at least 12 spines/setae of similar size and length to those on the endopod (figure 1*r*,*s*,*u*). A single spine occurs on the inner proximal-most margin of the precoxal area of the limb base (arrowed in figure 1*s*,*u*). Similar fine structures are probably present on the other trunk appendages and head appendages 4 and 5.

The mid-length axial part of each sternite is raised to form a low node (figure 1*p*); there is no evidence of a food groove. The posterior and especially the anterior limit of each sternite is marked by a transverse ridge (figure 1*p*). The abdomen is poorly preserved. There are possibly at least two additional (apodous) segments beyond which (figure 1*a*,*b*,*d*; dark brown) there appears to be mainly extraneous matter that masks the remainder of the trunk.

## 4. Discussion

### (a) Affinities and evolutionary significance

**Figure 2.**
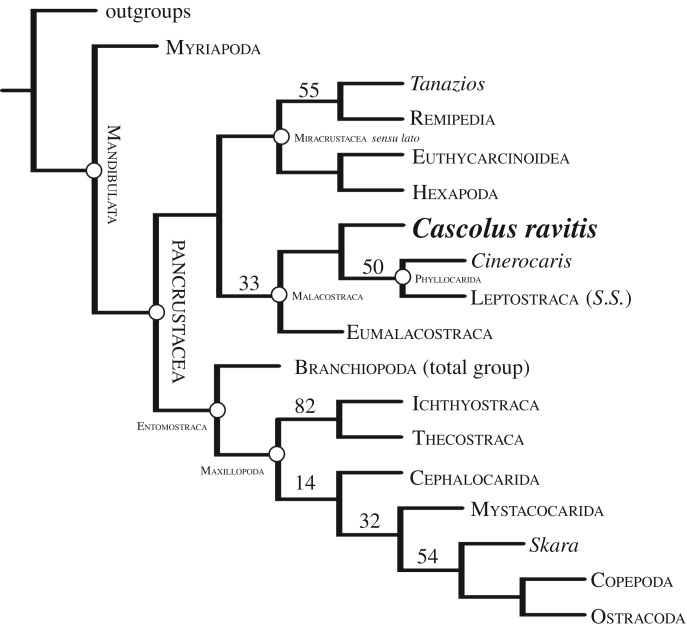
The phylogenetic position of *Cascolus ravitis*. Shown is a strict consensus of 13 most parsimonious trees of 143.67981 steps (CI = 0.509; RI = 0.868). Nodal support is given as GC values.

The combination of morphological characters in the new fossil is unknown in any other arthropod and it is assigned to a new genus. Phylogenetic analysis of a large database (see Material and methods) resolves *C. ravitis* as a stem-group leptostracan and sister to *Cinerocaris*+Leptostraca *s.s.* (figure 2; electronic supplementary material, figure S1). Phyllocarida (i.e. Archaeostraca + Leptostraca) is normally considered sister to Eumalacostraca [4,8], and together they constitute Malacostraca (figure 2) (see [8] for a review of alternative positions of Leptostraca). *Cascolus ravitis*, together with the associated Herefordshire Lagerstätte phyllocarid *Cinerocaris magnifica* [2,34], represent the oldest known members of crown-group Malacostraca. Only one purported fossil representative of Leptostraca, the Permian *Rhabdouraea*, has been reported [36].

*Cascolus ravitis* displays heteronomous post-antennulary appendages, which is a characteristic of mandibulates and evident in crustaceans [32,33,37–40]. The three long flagella of the antennule of *C. ravitis* resemble those in stomatopod malacostracans and also in the Cambrian stem mandibulate *Oelandocaris* [41], but the limb base of the *C. ravitis* antennule differs in lacking segmentation. The antennulary flagella also recall similar features in the first head appendage of the megacheirans *Leanchoilia* from the Cambrian [42–44] and *Enalikter* from the Silurian, but the *C. ravitis* antennule differs fundamentally from the first (‘great’) appendage of megacheirans in lacking a bipartite peduncle and ‘elbow joint’. Furthermore, the flagella of *C. ravitis* do not originate from individual spinose segments of a ramus as they do in *Leanchoilia.* More significantly, given where *C. ravitis* falls in the cladogram (figure 2), its antenna differs markedly from the antenna in Leptostraca in being biramous rather than uniramous (see [45]). The antenna in both the two well-preserved phyllocarids from the Devonian Hunsrück Slate, *Nahecaris stuertzi* [46] and *Oryctocaris balssi* [47], is also biramous. Thus, the evolution of the antenna in leptostracans is likely to have involved modification and reduction of the number of rami [35].

**Figure 3.**
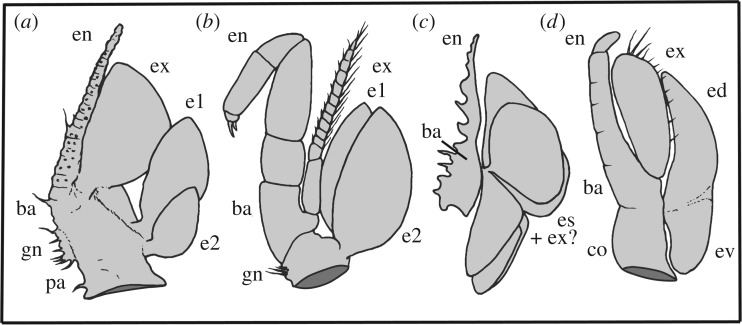
Representative malacostracans with trunk limbs that bear epipods. (*a*) Second trunk appendage of *Cascolus ravitis.* (*b*) Second pereopod of the Recent syncarid eumalacostracan *Anaspides tasmaniae* (redrawn, musculature omitted, from [32,33]). (*c*) Idealized reconstruction of the fifth thoracopod of the Silurian archaeostracan phyllocarid *Cinerocaris magnifica* (redrawn from [34], with interpretation after [33,34]). (*d*) Second pereopod of the Recent leptostracan phyllocarid *Nebalia pugettensis* (redrawn, musculature omitted, after [33,35]). Abbreviations as for figure 1, plus: co, coxa; ed, dorsal epipod; es, epipods; ev, ventral epipod.

Epipods, such as those on the rear head appendages and trunk appendages of *C. ravitis,* are known only in Eucrustacea (*sensu* [48]), where they occur in a few entomostracans (*sensu* [4]) and in Malacostraca. Appendages bearing two epipods, as in *C. ravitis*, are also present in some branchiopods, syncarids and in leptostracans [33,35], and at least two occur on appendages in the fossil *Cinerocaris* (figure 3). They may also be present in the Herefordshire Lagerstätte *Tanazios*, a putative stem-lineage crustacean [49], regarded as a labrophoran by Boxshall [33,50]. Cephalic epipods are rare, but one is present on the maxillule in copepods, and one occurs on the maxilla in myodocope ostracodes, where it is the only known example in this position in a living crustacean [33,35]*. Cascolus ravitis*, unlike *Cinerocaris*, has epipods on its cephalic limbs as well as those of the trunk.

The position of *C. ravitis* on the stem of Leptostraca is supported by the nature of the appendages at the rear of the head and in the trunk, with a paddle-like epipod and a multisegmented endopod with two rows of setae. *Cascolus ravitis* differs from other phyllocarids, including the living Leptostraca, in lacking a true carapace (that is, a head shield with a post-cephalic extension). However, the overall appearance of the cephalic shield (including ridges) and the presence of three flagella in the antennule are reminiscent of those features in stomatopods, which fall out at the base of the Eumalacostraca [4,8].

Malacostracans are arguably the only crustaceans with a tagmatized trunk [51]. Tagmosis in *C. ravitis* differs from that in other malacostracans, which have a thorax (pereon) of eight somites and an abdomen (pleon) of seven in phyllocarids, including leptostracans, and six in Eumalacostraca. *Cascolus ravitis*, by contrast, has nine appendage-bearing thorax somites, followed by an unknown number of apodous, presumably abdominal somites and perhaps a telson. The number of thoracic segments may be a late stage in the reduction towards the eight that are characteristic of Malacostraca from a plesiomorphic many-segmented trunk with serially similar limbs [52] as in basal Miracrustacea *s.l.* such as *Tanazios* and remipedes (in contrast, molecular analyses argue for a few-segmented ancestor state [53]). The presence of one fewer abdominal somites in Eumalacostraca than in Phyllocarida may be the result of a similar process. Such variation may reflect shifts in Hox gene expression along the trunk [51,54]. The phylogenetic position of *C. ravitis* implies that the eight-segmented thorax of Malacostraca may have been acquired convergently in Phyllocarida (including leptostracans) and in Eumalacostraca.

*Cascolus ravitis* demonstrates a number of plesiomorphic features similar to those in remipedes and cephalocarids, notably the similarity of the fourth and fifth head appendages to those of the trunk, and the lack of an articulated rostrum projecting anteriorly from the carapace. A movable rostrum is present in Leptostraca, some fossil phyllocarids and Stomatopoda, and its absence is potentially a plesiomorphic feature of Malacostraca. The presence of two epipods attached to the base of appendages in *C. ravitis* lends support to the presence of a multi-epipod condition (two in Malacostraca and possibly three in Entomostraca) in the pancrustacean ground plan [48], although Boxshall [32,33,50] pointed out the difficulty of establishing homologies between these structures, particularly in fossil taxa.

### (b) Mode of life

The Herefordshire biota lived within the Welsh depositional basin, probably in a slope setting at water depths of 100–200 m [9]. The remarkably preserved nature of *C. ravitis*, with no sign of distortion even of the extremely fine flagella, suggests that, like other animals of the Lagerstätte, it was preserved rapidly *in vivo*. The large paddle-shaped epipods and exopods may have functioned in locomotion and as respiratory (see [48]) and/or osmoregulatory organs. These laterally extensive structures recall the foliaceous epipods and exopods involved in respiration in, for example, the Recent leptostracans *Nebalia* and *Dahlella* [35,55,56]. Their relatively large surface area would presumably facilitate efficient oxygen uptake. The thick outer and inner borders and the intervening delicate cuticle of the epipods and exopods of *C. ravitis* draw close structural parallels with the epibranchial (efferent) and hypobranchial (afferent) canals and intervening thin tissue of gill lamellae of the living myodocopid ostracod *Leuroleberis*, and also with similar structures in living leptostracans, in which the canals function in transporting hemolymph in the respiratory–circulatory system [35,55]. The stout lateral margins of the triangular basal connection of each exopod of *C. ravitis* may also have housed circulatory canals and/or musculature.

The large stalked eyes of *C. ravitis* indicate an almost all-round field of view. As in most mandibulates the first head appendage of *C. ravitis* probably functioned as a sensory receptor of environmental conditions [32]. Extant Leptostraca comprises a few tens of mostly small marine species (5–15 mm long) found in intertidal settings and down to more than 2000 m depth. Most species occur in less than 200 m. The majority are epibenthic suspension feeders, but carnivorous scavengers and bathypelagic representatives are also known. The cephalized post-antennulary appendages of *C. ravitis* are clearly specialized for feeding and its gnathobases probably functioned to process and channel food to the mouth, although there is no obvious food groove. Like many of its arthropod faunal associates *C. ravitis* may have been nektobenthic.

## Supplementary Material

Electronic Supplementary Information Figure 1.

## Supplementary Material

Electronic Supplementary Information Figure 2.

## Supplementary Material

Electronic Supplementary Information Table 1.

## Supplementary Material

Electronic Supplementary Information Table 2.

## Data accessibility

The triangle-mesh model of holotype specimen OUMNH C.29698 is available in VAXML/STL format from the Dryad Digital Repository: http://dx.doi.org/10.5061/dryad.g1q8p [57]. Cladistic character-matrix datasets supporting this article have been uploaded as part of the electronic supplementary material.

## References

[1] Erwin DH, LaFlamme M, Tweedt SM, Sperling EA, Pisani D, Peterson KJ 2011 The Cambrian conundrum: early divergence and later ecological success in the early history of animals. Science 334, 1091–1096. (10.1126/science.1206375) 22116879

[2] Wolfe JM, Daley AC, Legg DA, Edgecombe GD 2016 Fossil calibrations for the arthropod tree of life. Earth Sci. Rev. 160, 43–110. (10.1016/j.earscirev.2016.06.008)

[3] Edgecombe GD 2010 Arthropod phylogeny: an overview from the perspective of morphology, molecular data and the fossil record. Arthr. Struct. Develop. 39, 74–87. (10.1016/j.asd.2009.10.002) 19854297

[4] Legg DA, Sutton MD, Edgecombe GD 2013 Arthropod fossil data increase congruence of morphological and molecular phylogenies. Nat. *Commun.* 5, 2485 (doi:10:1038/ncomms348) 10.1038/ncomms3485 24077329

[5] Edgecombe GD, Legg DA 2013 The arthropod fossil record. In Arthropod biology and evolution: *molecules, development, morphology* (eds Minelli A, Boxshall G, Fusco G), pp. 343–415. New York, NY: Springer.

[6] Edgecombe GD, Legg DA 2014 Origins and early evolution of arthropods. Palaeontology 57, 1–12. (10.1111/pala.12105)

[7] Oakley TH, Wolfe JM, Lindgren AR, Zaharoff AK 2012 Phylotranscriptomics to bring the understudied into the fold; monophyletic Ostracoda, fossil placement and pancrustacean phylogeny. Molec. Biol. Evol. 30, 215–233. (10.1093/molbev/mss216) 22977117

[8] Richter S, Scholtz G 2001 Phylogenetic analysis of the Malacostraca (Crustacea). J. Zool. Syst. Evol. Res. 39, 113–136. (10.1046/j.1439-0469.2001.00164.x)

[9] Briggs DEG, Siveter DJ, Siveter DJ 1996 Soft-bodied fossils from a Silurian volcaniclastic deposit. Nature 382, 248–250. (10.1038/382248a0)

[10] Briggs DEG, Siveter DJ, Siveter DJ, Sutton MD 2008 Virtual fossils from 425 million-year-old volcanic ash. Am. Sci. 96, 474–481. (10.1511/2008.75.474)

[11] Briggs DEG, Siveter DJ, Siveter DJ, Sutton MD, Garwood RJ, Legg D 2012 A Silurian horseshoe crab illuminates the evolution of chelicerate limbs. Proc. Natl Acad. Sci. USA 109, 15702–15705. (10.1073/pnas.1205875109) 22967511 PMC3465403

[12] Briggs DEG, Siveter DJ, Siveter DJ, Sutton MD, Legg D 2016 Tiny individuals attached to a new Silurian arthropod suggest a unique mode of brood care. Proc. Natl Acad. Sci. USA 113, 4410–4415. (10.1073/pnas.1600489113) 27044103 PMC4843443

[13] Briggs DEG, Siveter DJ, Siveter DJ, Sutton MD, Legg D 2016 Reply to Ross Piper: *Aquilonifer*‘s kites are not mites. Proc. Natl Acad. Sci. USA 113, E3320–E3321. (10.1073/pnas.1606265113) 27231220 PMC4914191

[14] Siveter DJ, Briggs DEG, Siveter DJ, Sutton MD 2010 An exceptionally preserved myodocopid ostracod from the Silurian of Herefordshire, UK. Proc. *R* *Soc* *B* 277, 1539–1544. (10.1098/rspb.2009.2122) PMC2871837 20106847

[15] Siveter DJ, Briggs DEG, Siveter DJ, Sutton MD, Joomun SC 2012 A Silurian myodocope with preserved soft-parts: cautioning the interpretation of the shell-based ostracod record. Proc. *R* *Soc* *B* 280, 20122664 (10.1098/rspb.2012.2664) PMC3574317 23235709

[16] Siveter DJ, Briggs DEG, Siveter DJ, Sutton MD, Legg D, Joomun SC 2014 A Silurian short-great-appendage arthropod. Proc. *R* *Soc* *B* 281, 20132986 (10.1098/rspb.2013.2986) PMC3906945 24452026

[17] Siveter DJ, Briggs DEG, Siveter DJ, Sutton MD 2015 A 425-million-year-old Silurian pentastomid parasitic on ostracods. Curr. Biol. 23, 1–6. (10.1016/j.cub.2015.04.035). 26004764

[18] Siveter DJ, Briggs DEG, Siveter DJ, Sutton MD, Legg D, Joomun SC 2015 *Enalikter* *aphson* is an arthropod: a reply to Struck *et al*. 2014. Proc. *R* *Soc* *B* 282, 20142663 (10.1098/rspb.2014.2663) PMC4375853 25716792

[19] Sutton MD, Briggs DEG, Siveter DJ, Siveter DJ 2011 A soft-bodied lophophorate from the Silurian of England. Biol. Lett. 7, 146–149. (10.1098/rsbl.2010.0540) 20685698 PMC3030883

[20] Sutton MD, Briggs DEG, Siveter DJ, Siveter DJ, Sigwart JD 2012 A Silurian armoured aplacophoran: implications for molluscan phylogeny. Nature 490, 94–97. (10.1038/nature11328) 23038472

[21] Orr PJ, Briggs DEG, Siveter DJ, Siveter DJ 2000 Three-dimensional preservation of a non-biomineralized arthropod in concretions in Silurian volcaniclastic rocks from Herefordshire, England. J. *Geol* *Soc* *Lond* 157, 173–186. (10.1144/jgs.157.1.173)

[22] Sutton MD, Briggs DEG, Siveter DJ, Siveter DJ 2001 Methodologies for the visualization and reconstruction of three-dimensional fossils from the Silurian Herefordshire Lagerstätte. Palaeont. Electron. 4, 1–17.

[23] Sutton MD, Garwood RJ, Siveter DJ, Siveter DJ 2012 SPIERS and VAXML: a software toolkit for tomographic visualisation, and a format for virtual specimen exchange. Palaeont. Electron. 15, p5T.

[24] Goloboff PA, Farris JS, Nixon KC 2008 TNT, a free program for phylogenetic analysis. Cladistics 24, 774–786. (10.1111/j.1096-0031.2008.00217.x)

[25] Nixon KC 1999 The Parsimony Ratchet, a new method for rapid parsimony analysis. Cladistics 15, 407–414. (10.1111/j.1096-0031.1999.tb00277.x) 34902938

[26] Goloboff PA 1999 Analysing large data sets in reasonable times: solutions for composite optima. Cladistics 15, 415–428. (10.1111/j.1096-0031.1999.tb00278.x) 34902941

[27] von Siebold CT 1848 Lehrbuch der vergleichenden Anatomie der Wirbellosen Thiere. In Lehrbuch der vergleichenden Anatomie (eds Cvon Siebold T, Stannius H), pp. 1–169. Berlin, Germany: von Veit and Co.

[28] Brunnich MT 1772 Zoologiae fundamenta praelectionibus academicis accomodata: Grunde i Dyrelaeren. Copenhagen, Denmark: Apud Frider. Crist. Pelt.

[29] Latreille PA 1802 Histoire naturelle générale et particulière des Crustacés et des Insectes, Familles naturelles des genres, 3rd edn Paris, France: Dupuis.

[30] Packard AS 1879 The nebaliad Crustacea as types of a new order. Ann. Mag. Nat. Hist. 3, 459 (10.1080/00222937908562419)

[31] Claus CFW 1880 Grundzuge der Zoologie, 4th edn Leipzig, Germany: NG Elwertsche.

[32] Boxshall GA 2004 The evolution of arthropod limbs. Biol. Rev. 9, 253–300. (10.1017/S1464793103006274) 15191225

[33] Boxshall GA 2013 Arthropod limbs and their development. In Arthropod biology and evolution: *molecules, development, morphology* (eds Minelli A, Boxshall G, Fusco G), pp. 241–267. London, UK: Springer.

[34] Briggs DEG, Sutton MD, Siveter DJ, Siveter DJ 2004 A new phyllocarid (Crustacea: Malacostraca) from the Silurian Fossil Lagerstätte of Herefordshire, UK. Proc. *R* *Soc* *B* 271, 131–138. (10.1098/rspb.2003.2593) PMC1691579 15058388

[35] Boxshall GA, Jaume D 2009 Exopodites, epipodites and gills in crustaceans. Arthrop. System. Phylog. 67, 229–254.

[36] Schram F, Malzahn E 1984 The fossil leptostracan *Rhabdouraea bentzi* (Malzahn, 1958). Trans San Diego Soc. Nat. Hist. 20, 95–98. (10.5962/bhl.part.29000)

[37] Waloszek D 1993 The Upper Cambrian *Rehbachiella* and the phylogeny of Branchiopoda and Crustacea. Fossils and Strata 32, 1–202. (10.1111/j.1502-3931.1993.tb01537.x)

[38] Walossek D 1999 On the Cambrian diversity of Crustacea. In Crustaceans and the Biodiversity Crisis, vol. 1 (eds Schram FR, von Vaupel Klein JC), pp. 3–27. Leiden, The Netherlands: Brill.

[39] Waloszek D 2003 Cambrian ‘Orsten’-type preserved arthropods and the phylogeny of Crustacea. In The new panorama of animal evolution (eds Legakis A, Sfenthourakis S, Polymeni R, Thessalou-Legaki M), pp. 66–84. Moscow, Russia: Pensoft Publishers.

[40] Waloszek D, Maas A, Chen J-Y, Stein M 2007 Evolution of cephalic feeding structures and the phylogeny of Arthropoda. Palaeogeo. Palaeoclim. Palaeoecol. 254, 273–287. (10.1016/j.palaeo.2007.03.027)

[41] Stein M, Waloszek D, Maas A, Haug JT, Müller KJ 2008 The stem crustacean *Oelandocaris oelandica* re-visited. Acta Pal. Polon. 53, 461–484. (10.4202/app.2008.0308)

[42] Liu Yu, Hou X-G, Bergström J 2007 Chengjiang arthropod *Leanchoilia illecebrosa* (Hou, 1987) reconsidered. Geo. Fören. Stock. För. 129, 263–272.

[43] Edgecombe GD, García-Bellido CD, Paterson JR 2011 A new leanchoiliid megacheiran arthropod from the lower Cambrian Emu Bay Shale, South Austrailia. Acta Pal. Pol. 56, 385–400. (10.4202/app.2010.0080)

[44] Haug JT, Briggs DEG, Haug C 2012 Morphology and function in the Cambrian Burgess Shale megacheiran arthropod *Leanchoilia superlata* and the application of a descriptive matrix. BMC Evol. Biol. 12, 162 (10.1186/1471-2148-12-162) 22935076 PMC3468406

[45] Walker-Smith GK, Poore GCB 2001 A phylogeny of the Leptostraca (Crustacea) with keys to families and genera. Mem. Mus. Victoria 58, 383–410.

[46] Bergström J, Briggs DEG, Dahl E, Rolfe WDI, Stürmer W 1987 *Nahecaris stuertzi*, a phyllocarid crustacean from the Lower Devonian Hunsrück Slate. Pal. Zeit. 61, 273–298. (10.1007/BF02985909)

[47] Bergmann A, Rust J 2014 Morphology, palaeobiology and phylogeny of *Oryctocaris balssi* gen. nov. (Arthropoda), a phyllocarid from the Lower Devonian Hunsrück Slate (Germany). J. Syst. Palaeont. 12, 427–444. (10.1080/14772019.2012.750630)

[48] Maas A, Haug C, Haug JT, Olesen J, Zhang X-G, Waloszek D 2009 Early crustacean evolution and the appearance of epipodites and gills. Arthrop. System. Phylog. 67, 255–273.

[49] Siveter DJ, Sutton MD, Briggs DEG, Siveter DJ 2007 A new probable stem lineage crustacean with three-dimensionally preserved soft-parts from the Herefordshire (Silurian) Lagerstätte, UK. Proc. *R* *Soc* *B* 274, 2099–2107. (10.1098/rspb.2007.0429) PMC2706188 17609185

[50] Boxshall GA 2007 Crustacean classification: on-going controversies and unresolved problems. Zootaxa 1668, 313–325.

[51] Abzhanov A, Kaufman TC 2000 Crustacean (malacostracan) Hox genes and the evolution of the arthropod trunk. Development 127, 2239–2249. 10804167 10.1242/dev.127.11.2239

[52] Olesen J, Walossek D 2000 Limb ontogeny and trunk segmentation in *Nebalia* species (Crustacea, Malacostraca, Leptostraca). Zoomorphology 120, 47–64. (10.1007/s004350000024)

[53] Regier JC, Shultz JW, Zwick A, Hussey A, Ball B, Wetzer R, Martin JW, Cunningham CW 2010 Arthropod relationships revealed by phylogenomic analysis of nuclear protein-coding sequences. Nature 463, 1079–1083. (10.1038/nature08742) 20147900

[54] Martin A, Serano JM, Jarvis E, Bruce HS, Wang J, Ray S, Barker CA, O'Connell LC, Patel NH 2016 Crisp/Cas9 mutagenesis reveals versatile roles of Hox genes in crustacean limb specification and evolution. Curr. Biol. 26, 14–26. (10.1016/j.cub.2015.11.021) 26687626

[55] Vannier J, Abe K, Ikuta K 1996 Gills of cylindroleberid ostracodes exemplified by *Leuroleberis surugaensis* from Japan. J. Crust. Biol. 16, 453–468. (10.2307/1548735)

[56] Shu D-G, Vannier J, Luo H-L, Chen L, Zhang X-L, Hu S-X 1999 Anatomy and lifestyle of *Kunmingella* (Arthropoda, Bradoriida) from the Chengjiang fossil Lagerstätte (lower Cambrian; southwest China). Lethaia 32, 279–298. (10.1111/j.1502-3931.1999.tb00547.x)

[57] Siveter DJ, Briggs DEG, Siveter DJ, Sutton MD, Legg D 2017 Data from: A new crustacean from the Herefordshire (Silurian) Lagerstätte, UK, and its significance in malacostracan evolution. Dryad Digital Repository. (10.5061/dryad.g1q8p) PMC5378094 28330926

