# Supplementary figures and images for: A new crustacean from the Herefordshire (Silurian) Lagerstätte, UK, and its significance in malacostracan evolution
Source: Proc Biol Sci. 2017 Mar 22;284(1851):20170279. doi: 10.1098/rspb.2017.0279 (PMC5378094; doi:10.1098/rspb.2017.0279)

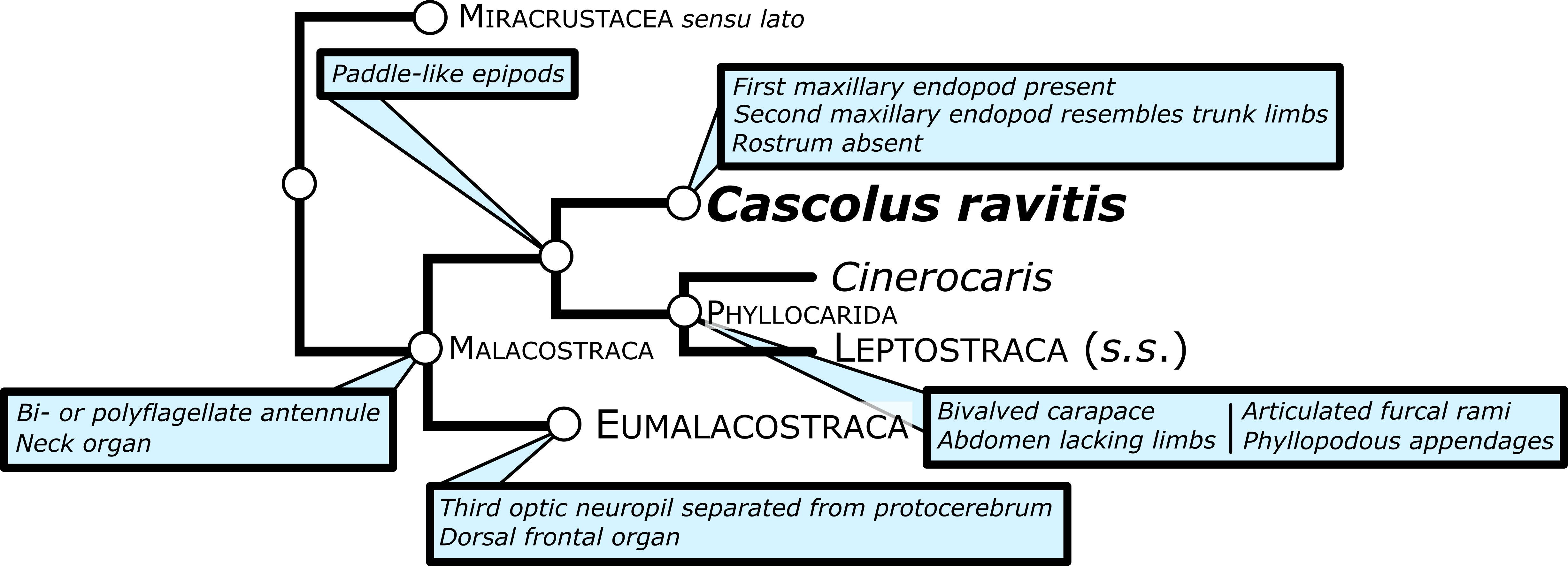

Supplement: Electronic Supplementary Information Figure 1. [file rspb20170279supp1.jpg]

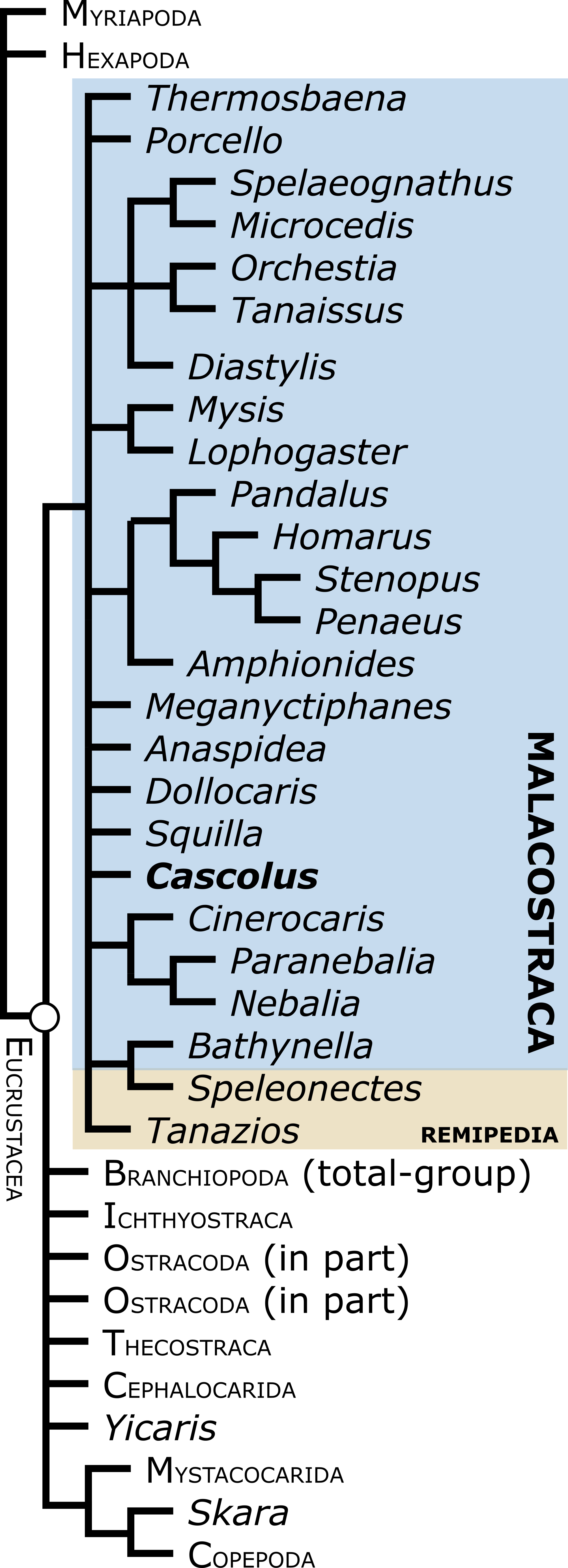

Supplement: Electronic Supplementary Information Figure 2. [file rspb20170279supp2.jpg]
