# Supplementary material for: A new crustacean from the Herefordshire (Silurian) Lagerstätte, UK, and its significance in malacostracan evolution
Source: Proc Biol Sci. 2017 Mar 22;284(1851):20170279. doi: 10.1098/rspb.2017.0279 (PMC5378094; doi:10.1098/rspb.2017.0279)
Supplement: Electronic Supplementary Information Table 2. [file rspb20170279supp4.pdf]

Supplementary Information

Morphology

1. *Trunk annuli: (0) absent, (1) present (coding for the external, cuticular expression of segmentation)*. **[R2011:18]**
2. *Tegumental annulations: (0) absent, (1) present*. **[R2011:19]**
3. *Sclerotization of cuticle into hard, articulated tergal exoskeleton: (0) absent, (1) present*. **[R2011:20]**
4. *Cuticle mineralization: (0) absent, (1) present, calcite, (2) present, calcium phosphate*. **[R2011:21]**
5. *Tendon cells with tonofilaments (keratin structures involved in cell-cell adhesion) penetrating epidermis: (0) absent, (1) present*. **[R2011:23]**
6. *Dorsal longitudinal ecdysial suture with forking on head: (0) absent, (1) present*. **[R2011: 24]**
7. *Transverse and antennocellar sutures on head shield: (0) absent, (1) present*. **[R2011:25]**
8. *Resilin protein: (0) absent, (1) present*. **[R2011:26]**
9. *Moulting glands: (0) absent, (1) present*. **[R2011:27]**
10. *Distinct head: (0) absent, (1) present*. **[M2009:25]**
11. *Anterior margin of cephalon notched: (0) absent, (1) present*. **[O2013:28]**
12. *Anterior sclerite associated with ocular segment: (0) absent, (1) present*. **[D2009:14]**
13. *Frontal organs on prehypostomal sclerite: (0) absent, (1) present*. **[P2010:11]**
14. *Tergal covering of all head segments fused as cephalic shield: (0) absent, (1) present*. **[B2002:3; R2011:83]**
15. *Subtrapezoidal cephalic shield restricted to anterior-most head segments: (0) absent, (1) present*. **[O2013:77]**
16. *Elevated marginal rim: (0) absent, (1) present*. **[O2013:33]**
17. *Anterior glossate process: (0) absent, (1) present*. See ref. 24 (ch.17).
18. *Anterior end of prosoma with medial marginal or submarginal pointed process: (0) absent, (1) present*. **[S2007:3]**
19. *Anterolateral spines: (0) absent, (1) present*. **[R2012:2]**
20. *Mediolateral spines: (0) absent, (1) present*. **[R2012:3]**
21. *Secondary spines on cephalic shield: (0) absent, (1) present*. **[R2012:5]**
22. *Free head shield: (0) absent, (1) present*. **[O2013:25]**
23. *Bivalved carapace: (0) absent, (1) present*. **[B2002:4]**
24. *Head capsule: (0) absent, (1) present*. See ref. 24 (ch.24).
25. *Cephalic doublure: (0) absent, (1) present*. **[O2013: 27]**
26. *Width of doublure: (0) narrow to moderately wide, (1) wide, (2) covers entire ventral side of cephalon.* **[O2013:78]**
27. *Cephalic tagmosis (number of limb-bearing segments): (0) two limb-bearing segments, (1) three limb-bearing segments, (2) four limb-bearing segments, (3) five limb-bearing segments, (4) cephalosoma with four post-pedipalpal locomotory limbs: (5) six limb-bearing segments: (6) seven limb-bearing segments.***[***cf***. R2011:84]**

28. *Transverse furrows on prosomal carapace corresponding to margins of segmental tergites: (0) absent, (1) present*. **[R2011:86]**

29. *Cephalic shield with distinct propeltidium: (0) absent, (1) present*. **[S2007:6]**
30. *Mesopeltidium divided medially by a shallow sulcus: (0) absent, (1) present*.
31. *Sejugal furrow: (0) absent, (1) present*. **[S2007:7]**
32. *Cardiac lobe: (0) absent, (1) present*. **[R2011:87]**
33. *Glabellar furrows or lobes: (0) absent, (1) present*. **[O2013:35]**
34. *Cephalic kinesis: (0) absent, (1) present*. **[R2011:88]**
35. *Carapace adductor: (0) absent, (1) present*. **[R2011:89]**
36. *Carapace growth lines: (0) absent, (1) present*. **[R2011:90]**
37. *Flattened head capsule, with head bent posterior to the clypeus, accommodating antennae at anterior margin of head: (0) absent, (1) present*. **[R2011:91]**
38. *Clypeofrontal sulcus (epistomal suture): (0) absent, (1) present*. **[R2011:92]**
39. *Genal spines (posterior corners of head shield extended): (0) absent, (1) present, short (2) present (as long as trunk)*. **[***cf*. **R2011:93]**
40. *Complete post occipital ridge on head: (0) absent, (1) present*. **[R2011:188]**
41. *Collum covering posterior part of head capsule and part of segment II: (0) absent, (1) present*. **[R2011:211]**
42. *Cephalic articulation fused: (0) absent, (1) present*. **[O2013:46]**
43. *Cephalic shield overlapping anterior trunk tergite(s): (0) overlap similar to that between adjacent thoracic segments, (1) partial overlap on anterior trunk segment (2) cephalic shield covers multiple anterior tergites*.
44. *Cephalic shield articulates with reduced trunk tergite: (0) absent, (1) present*. **[O2013:48]**
45. *Prosoma (cephalic shield)-opisthosoma (trunk) coupling mechanism: (0) absent, (1) present.* **[G2002:24]**
46. *Post-oral plate: (0) absent, (1) present*. **[G2002:17]**
47. *Sternum: (0) divided, (1) undivided*. This character refers to the ventral cephalic plates of chelicerates and is distinct from the sternum of crustaceans. **[G2002:18]**
48. *Sternum shape: (0) triangular, (1) pentagonal, (2) small and circular (3) ovoid*.
49. *V-shaped medial sulcus on sternum: (0) absent, (1) present*. This character refers to the sternum of scorpions only. Extant scorpions lack a V-shaped sulcus, whereas Carboniferous scorpions tend to have a pronounced sulcus. The sternum of Silurian scorpions, e.g. *Proscorpius*, is undivided, and hence this character is coded as absent in these taxa. **[L2012:49]**
50. *Posteromedial sulcus in sternum: (0) absent, (1) present*. This character is similar to ch. 49 as most taxa with a V-shaped sulcus also possess a poteromedial sulcus, but it is also possible to find this character in isolation and therefore the two states were coded as separate characters. **[L2012:50]**
51. *Medial intercoxal region: (0) all pedal coxae separated medially, (1) anterior pedal abutting medially, posterior coxae separated, (1) anterior pedal coxae separated medially, (2) anterior pedal coxae separated medially, posterior coxae abutting, (3) all pedal coxae abutting medially, (4) epimera: coxae undifferentiated from ventral body wall*. **[S2007:17]**

52. *Trunk narrows anteriorly relative to cephalic shield, widest posteriorly: (0) absent, (1) present*. **[O2013:49]**
53. *Trunk somites divided into distinct tergites and sternites: (0) absent, (1) present*. This character is coded for taxa which show evidence of demarcation between the dorsal tergites and ventral somites, such as *Nereocaris*, and is therefore distinct from ch. 93 which is restricted to taxa that have undergone fusion of the tergite, sternites and pleurites into a single unit. **[L2012:53]**
54. *Boundaries of anterior trunk segments reflexed anterolaterally: (0) absent, boundaries transverse or reflexed posterolaterally: (1) present.* **[O2013:50]**
55. *Lateral flaps (body extends laterally into imbricated, unsclerotised flaps): (0) absent, (1) present*. **[D2009:36; R2011:262]**
56. *Longitudinal ("gill-like") wrinkling on lateral part of flaps: (0) absent, (1) present*. **[D2009:38; R2011:263]**
57. *Free thoracic tergites: (0) absent, (1) present*. **[O2013:42]**
58. *Decoupling of thoracic tergites and segments: (0) absent, (1) present*. **[O2013:43]**
59. *Tergite articulations: (0) tergites non-overlapping, (1) extensive overlap of tergites, (2) edge-to-edge pleural articulations*. **[O2013:44]**
60. *Tergal scutes extend laterally into paratergal folds: (0) absent, (1) present*. **[R2011:264]**
61. *Trunk elongate (consisting of >25 somites): (0) absent, (1) present.*
62. *Radial arrangement of tergal pleurae: (0) absent, (1) present*. **[O2013:53]**
63. *Trunk tergites with serrate lateral margin: (0) absent, (1) present*. **[E2011:17]**
64. *Paired tergal carinae: (0) absent, (1) present*. **[E2011:20]**
65. *Trunk effacement: (0) trunk with defined (separate or fused) tergite boundaries: () trunk tergite boundaries effaced laterally, (2) trunk tergite boundaries completely effaced*. **[O2013:45]**
66. *Joints between posterior tergites functional, anterior ones variably fused: (0) absent, (1) present.* **[O2013:51]**
67. *Posterior tergite bearing axial spine: (0) absent, (1) present*. **[O2013:52]**
68. *Anterior tergal processes: (0) absent, (1) present*. **[O2013:55]**
69. *Fusion of paratergal folds into a dorsal shield: (0) absent, (1) present*. **[R2012:1]**
70. *Relative length of thorax: (0) longer than pygidium, (1) shorter than pygidium*. **[O2013:79]**
71. *Reduced thorax: (0) absent, (1) present*. A reduced thorax, composed of five or fewer segments, is prevalent amongst nektaspids, particularly liwiids, *Panlongia* and *Campanamuta*. This character is only applicable to taxa with a pygidium (ch. 109). **[L2012:71]**
72. *Articulating half ring on thoracic segments: (0) absent, (1) present*. **[O2013:80]**
73. *Raised axial region: (0) absent, (1) present*.
74. *Axial furrows: (0) absent, (1) present*. **[O2013:81]**

75. *Number of opisthosomal somites: (0) five, (1) eight, (2) nine, (3) 10, (4) 11, (5) 12, (6) 13.* **[S2007:95]**
76. *Fusion of tergites of postoral somites VIII and IX: (0) absent, (1) present*. **[S2007:100]**
77. *First mesosomal somite: (0) shorter than, or equal in length to second mesosomal somite, (1) longer than second mesosomal somite*.
78. *Anterior transverse ridge on mesosomal tergites: (0) absent, (1) present*.
79. *Opisthosoma divided longitudinally: (0) absent, (1) present*. **[S2007:115]**
80. *Fusion of all (opisthosomal) tergites behind the opercular tergite into the thoracetron: (0) absent, (1) present*. **[R2011:240]**
81. *Opisthosoma greatly reduced, forming a slender tube emerging from behind the posteriormost legs, with a terminal anus: (0) absent, (1) present*. **[R2011:241]**
82. *Opisthosoma consisting of three somites or less: (0) absent, (1) present*. This character (and ch. 83) refers to pycnogonids only.
83. *Opisthosoma comprising a single somite, without a telson: (0) absent, (1) present*.
84. *Demarcation of opisthosomal somites: (0) absent, (1) present*. In extant pycnogonids the opisthosoma is greatly reduced and has lost evidence of original segmentation. **[L2012:84]**
85. *Diplosegments: (0) absent, (1) present*. **[R2011:258]**
86. *Paramedian sutures: (0) absent, (1) present*. **[R2011:265]**
87. *Intercalary sclerites: (0) absent, (1) developed as small rings, (2) developed as pretergite and presternite*. **[R2011:266]**
88. *Trunk heterotergy: (0) absent, (1) present (alternating long and short tergites, with reversal of lengths between seventh and eighth walking leg-bearing segments)*. **[R2011:267]**
89. *Trunk sternites: (0) large sternum, (1) sternal area divided into two hemisternites by linea ventralis, (2) sternum mostly membranous, with pair of small sternites, (3) sternal plate bears Y-shaped ridge/apodeme, (4) sternites extended rearwards to form substernal laminae, (5) thoracic sternal areas reduced and partly invaginated along the median line, (6) sternal plate absent*. **[R2011:268]**
90. *Pleural part of trunk segments: (0) pleurites absent, (1) supracoxal arches (catapleural and anapleural arches) on each segment, (2) pleural part of thoracic segments II and III consisting of a single sclerite with a large pleural process, (3) pleuron in each thoracic segment composed of a single sclerite divided into anterior and posterior parts by pleural suture, from which a pleural apophysis is invaginated, its internal end connected to the furcal arm*. **[R2011:270]**
91. *Procoxal and metacoxal pleurites surrounding coxa: (0) pleurites absent or incompletely surrounding coxa, (1) procoxa and metacoxa surround coxa*. **[R2011:271]**
92. *Pleuron filled with small pleurites: (0) absent, (1) present*. **[R2011:273]**

93. *Complete body ring: (0) absent (sternites and/or pleurites free), (1) present (sternites, pleurites and tergites fused)*. **[R2011:274]**
94. *Width of first opisthosomal segment: (0) broad, (1) narrow, developed as pedicel*. **[R2011:250]**
95. *Pereion tagmosis: (0) one locomotory tagma, (1) two locomotory tagmata*. **[R2011:253]**
96. *Thorax with three-limb bearing segments: (0) not differentiated, (1) three*. This character refers to the hexapod thorax only. **[R2011:254]**
97. *Abdomen differentiated as limb-free somites: (0) absent, (1) present*. In extant arthropods this is posterior to expression domain of Ubx, abdA and abdB. **[R2011:252;** *cf*. **O2013:59]**
98. *Abdomen as posterior differentiated segments (0) absent, (1) present*. **[O2013:60]**
99. *Length of abdomen: (0) 1 segment, (1) 2 segments, (2) 3 segments, (3) 5 segments*. **[O2013:59]**
100. *Spines on posterior margin of abdominal somites: (0) absent, (1) present*. The abdominal somites of *Canadaspis*, *Perspicaris* and *Loricicaris* possess rows of spines along their posterior margin. **[L2012:100]**
101. *Abdominal segmentation (in hexapods): (0) six segments, (1) ten segments, (2) eleven segments, (3) twelve segments*. **[R2011:316]**
102. *Segmentation of pleon: (0) seven segments, (1) six segments*. **[R2011:257]**
103. *Trunk bipartite with narrow posterior: (0) absent, (1) present*. The narrow posterior tagma of the fuxianhuiids *Fuxianhuia* and *Guangweicaris* is differentiated from the anterior somites, which possess extensive lateral folds **[L2012:103]**
104. *Caudal metasoma: (0) absent, (1) present*. In scorpions, some chasmataspids, and some eurypterids the metasoma is caudal. **[L2012:104]**
105. *Number of metasomal somites: (0) zero, (1) two, (2) three, (3) five, (4) nine*. **[S2007:116]**
106. *Carinae on metasoma: (0) absent, (1) present*. **[L2012:106]**
107. *Elongate coxopleurites on anal legs: (0) absent, (1) present*. **[R2011:272]**
108. *Posterior tergites strongly curved in dorsal aspect compared to anterior tergites: (0) absent, (1) present*. **[O2013:61]**
109. *Pygidium: (0) absent, (1) present*. **[C2004:47; E2011:21]**
110. *Pygidium with medial keel: (0) absent, (1) present*. **[O2013:64]**
111. *Pygidium with broad-based medial spine: (0) absent, (1) present*. **[O2013:65]**
112. *Pygidium with lateral spines: (0) absent, (1) present*. **[O2013:66]**
113. *Size of pygidium: (0) shorter to slightly longer than cephalon, (1) much longer than cephalon*. **[P2010:23]**
114. *Posterior tagmata with elongate lateral processes: (0) absent, (1) present*. This character is scored as present for both the lateral posterior processes of dinocaridids and bivalved stem-arthropods. The flap-like lateral processes of *Fortiforceps* and *Shankouia* are also coded as homologous.

115. *Posterior tagmata composed of three paired tail flaps: (0) absent, (1) present*. **[R2011:317]**
116. *Lateral telson processes fused: (0) absent, (1) present*. The telsons of *Jugatacaris*, *Pectocaris* and *Odaraia* possess lateral processes which are fused together but show evidence of original segmentation. **[L2012:116]**
117. *Posterior most tagma modified into a fluke: (0) absent, (1) present*.
118. *Medial telson process: (0) absent, (1) present*. *Nereocaris*, *Jugatacaris*, *Pectocaris* and *Odaraia* possess a dorso-medial telson process. In *Odaraia* this process is fused to the other telson elements. **[L2012:118]**
119. *Lateral telson processes spinose: (0) absent, (1) present*. **[L2012:119]**
120. *Lateral telson processes foliate: (0) absent, (1) present*. The lateral telson processes of *Perspicaris* and *Canadaspis* are leaf-shaped with a crenulated or spinose margin. **[L2012:120]**
121. *Lateral telson processes recurved: (0) absent, (1) present*.
122. *Lateral telson processes with lanceolate tips: (0) absent, (1) present*.
123. *Telson processes flap-shaped: (0) absent, (1) present.* The lateral telson processes of *Fortiforceps* and the fuxianhuiids *Shankouia* and *Liangwangshania* are modified into paddle-shaped flaps. **[L2012:123]**
124. *Annulated caudal filament: (0) absent, (1) present*. **[R2011:318]**
125. *Abdominal segment XI modified as cerci: (0) absent, (1) present*. **[R2011:319]**
126. *Articulate furcal rami: (0) absent, (1) present*. **[R2011:320]**
127. *Uropods: (0) absent, (1) present*. **[R2011:321]**
128. *Paired flaps lateral to telson: (0) absent, (1) present*. **[E1999:25; O2013:75]**
129. *Nature of lateral telson flaps: (0) "uropods", (1) furca*. **[O2013:76]**
130. *Pre-telson somite elongate and rigid: (0) absent, (1) present*. The pre-telson somite of *Pectocaris* and *Jugatacaris* are elongate, equivalent in length to three or more abdominal somites. **[L2012:130]**
131. *Telson: (0) absent, (1) present*.
132. *Telson shape: (0) round, (1) laterally depressed, (2) dorso-ventrally depressed*. **[R2011:323]**
133. *Telson shape II: (0) styliform, (1) paddle-shaped, (2) cap-like, (3) lanceolate, (4) flagellate, (5) with at least two elongate lateral processes/spines.* **[***cf*. **O2013:68]**
134. *Telson curved dorsally: (0) absent, (1) dorsally*.
135. *Length of telson: (0) shorter than half the length of the trunk, (1) longer than half the length of the trunk*. **[O2013:70]**
136. *Segmentation of telson: (0) absent (unjointed), present*. **[P2010:28]**
137. *One pair of dorsal telsonal setae: (0) absent, (1) present*. **[R2011:324]**
138. *Telson fringed with setae: (0) absent, (1) present*. **[E2011:24]**
139. *Paired terminal spinnerets: (0) absent, (1) present*. **[R2011:326]**
140. *Anal segment with a pair of large sense calicles, each with a long sensory seta: (0) absent, (1) present*. **[R2011:327]**
141. *Paired segmental ventrolateral appendages: (0) absent, (1) present*.
142. *Segmental appendage musculature: (0) absent, (1) present*.

143. *Orientation of the anteriormost limbs: (0) lateral, (1) ventral*.
144. *Anterior appendages differentiated from posterior trunk appendages: (0) absent, (1) present*.
145. *Frontal appendage with row(s) of elongate spines along inner margin: (0) absent, (1) present*. **[R2011:146]**
146. *Frontal appendages attached or fused basally: (0) separated, (1) basally attached or fused*. **[M2009:6; R2011:147]**
147. *Lobopods with pads and claws: (0) absent, (1) present*. **[R2011:276]**
148. *Strongly sclerotised, articulated limbs with pivot joints and intrinsic muscles: (0) absent, (1) present*. **[R2011:277]**
149. *Sclerotisation and arthropodisation of cephalic appendages: (0) absent, (1) present*. **[L2012:149]**
150. *Sclerotisation and arthropodisation of trunk appendages: (0) absent, (1) present*.
151. *Biramy in post-antennal limbs: (0) absent, (1) present*. **[R2011:278]**
152. *Post-antennal limb rami stemming from a protopodite that is rigid, antero-posteriorly flattened, bearing spines/setae along its inner edge: (0) absent (protopodite undifferentiated from limb stem), (1) present*. **[R2011:279]**
153. *Protopodite of post-mandibular limbs elongate, fleshy, extended as numerous soft, setiferous endites: (0) absent, (1) present*. **[R2011:280]**
154. *Coxopodite articulation: (0) arthrodial membrane only, (1) pleural condyle, (2) sternal condyle, (3) sternal and pleural condyle, (4) internal plate*. **[R2011:288]**
155. *Exopods of second antennal and mandibular (where exopod present) segments multi-annular with inwardly directed spines, including laval/juvenile stages: (0) absent, (1) present*. **[R2011:283]**
156. *Exopods fringed by strong setae: (0) setae absent, (1) strong, cylindrical or flattened setae present*. **[R2011:284]**
157. *Bilobate exopod shaft (proximal lobe hinged along length of basipodite, bearing imbricated lamellar setae; distal lobe fringe of short setae): (0) absent (exopod is an undivided flap), (1) present*. **[R2011:285]**
158. *Proximal section of exopod: (0) lobe, (1) shaft*. **[C2004:14; P2010:33]**
159. *Distal lobe of exopod: (0) small to moderate sized flap, (1) large, tear-drop shaped lobe with long attachment.* **[O2013:12]**
160. *Divided exopod with septum: (0) absent, (1) present*. **[O2013:13]**
161. *Imbricated exopod lamellae: (0) absent, (1) present*. **[O2013:14]**
162. *Exopod lamellae: (0) thick, flat, (1) delicate, comb-like*. **[O2013:15]**
163. *Shape of "simple" exopods: (0) rounded, (1) triangular, (3) with lamellae.*
164. *Differentiation of setae along exopod: (0) uniform or gradationally variable setae along length of exopod, (1) lamellar setae confined to proximal lobe, with distal lobe bearing slender setae*. **[E2011:30]**
165. *Exopod shaft consisting of numerous segments each bearing a single seta/filament: (0) absent, (1) present*.
166. *Pre-"great-appendage" appendages: (0) absent, (1) present*.

167. *Frontal filaments: (0) absent, (1) present*. **[R2011:148]**
168. *Appendage of second (deutocerebral) head segment: (0) locomotory leg 1, (1) antenna (antennula in crustaceans), (2) chelicerae / chelifore , (3) jaw*. **[R2011:149]**
169. *Antennal rami: (0) uniramous, (1) polyramous, (2) one ramus + scale*. **[R2011:150]**
170. *Antennule lobate, divided along most of its length into sensilla-bearing lobes: (0) absent (non-lobate antenna/first antenna), (1) present*. **[R2011:151]**
171. *Antennular sensilla: (0) sensilla present on non-terminal part of antennule (antenna in myriapods and hexapods), (1) confined to tip*. **[R2011:152]**
172. *Antennal apical cone sensilla: (0) absent, (1) present*. **[R2011:153]**
173. *Two lateral areas bearing club-like sensilla on terminal antennal article: (0) absent, (1) present*. **[R2011:154]**
174. *Intrinsic muscles of antennae: (0) absent, (1) present*. **[2011:155]**
175. *Scape and pedicel differentiated, with Johnson's organ: (0) absent, (1) present*. **[R2011:156]**
176. *Antennal circulatory vessels: (0) joined, (1) separate, (2) absent*. **[R2011:157]**
177. *Ampullo-ampullary dilator and ampullo-aortic dilator: (0) absent, (1) present*. **[R2011:158]**
178. *Stratocyst in basal segment of first antenna: (0) absent, (1) present*. **[R2011:159]**
179. *Cheliceral segmentation: (0) three segments or more, (1) two segments.* **[***cf.* **R2011:160]**
180. *Number of spine-bearing articles on chelate appendage: (0) three+moveable finger, (1) two+moveable finger, (2) one+moveable finger; (3) moveable finger*. **[E2011:5]**
181. *Long spinose projections on distal part of terminal three podomeres of chelate appendage bearing flagellum: (0) absent, (1) present.* **[E2011:6]**
182. *Length of flagellate great-appendage: (0) flagellae terminate at midlength of trunk, (1) flagellae extending to end of body*. **[E2011:7]**
183. *Plagula ventralis: (0) absent, (1) present*. **[R2011:161]**
184. *Chelicerocarapacal articulation: (0) absent, (1) present*. **[S2007:20]**
185. *Cheliceral tergo-deuteromerite muscle: (0) absent, (1) present*. **[R2011:162]**
186. *Naked cheliceral fang: () absent, (1) present*. The chelicerae of most chelicerates are covered in a layer of setae, but in spiders (Araneae) these setae are lacking. **[L2012:186]**
187. *Cheliceral venom gland: (0) absent, (1) present*. **[S2007:27]**
188. *Appendage on third (tritocerebral) head segment: (0) unspecialised locomotory limb, (1) second antenna, (2) absent (intercalary segment), (3) pedipalp, (4) oral papilla with slime glands and adhesive glands, (5) subchelate appendage,(6) "swimming paddle"*. **[R2011:163]**
189. *Single segmented antennal scale: (0) absent, (1) present*. **[R2011:164]**
190. *Antennal naupliar protopod: (0) short, (1) long*. **[R2011:165]**
191. *Palpal coxae fused ventromedially: (0) absent, (1) present*. **[S2007:30]**

192. *Rostrosoma: (0) absent, (1) present*. **[S2007:33]**
193. *Pedipalp (or equivalent) limb morphology: (0) pediform, (1) raptorial, (2) chelate (non-scorpion-type), (3) chelate (scorpion-type)*. **[***cf.* **R2011:166]**
194. *Pedipalp patella: (0) shorter than, or equal in length to manus, (1) longer than manus.*
195. *Prominent carina on pedipalp patella: (0) absent, (1) present*.
196. *Palpal apotele: (0) absent, (1) present*. **[S2007:47]**
197. *Raptorial second (tritocerebral) appendages: (0) absent, (1) present*.
198. *Exopod on tritocerebral segment much longer than endopod: (0) absent, (1) present*.
199. *Oviger: (0) absent, (1) present*. **[R2011:189]**
200. *Largest podomere of oviger: (0) first, (1) second, (3) sixth*.
201. *Walking leg I antenniform: (0) absent, (1) present*.
202. *Walking leg III and IV with femur shorter than patella and with principle site of flexion/extension at patella-tibia joint: (0) absent, (1) present*. **[S2007:48]**
203. *Walking leg II longer than adjacent leg and modified as feeler: (0) absent, (1) present*. **[R2011:190]**
204. *Distal ends of walking legs flattened: (0) absent, (1) present*.
205. *Coxal swing: (0) absent, (1) present*. **[R2011:287]**
206. *Musculi lateralis: (0) absent, (1) present*. **[R2011:292]**
207. *Coxotrochanteral joint: (0) simple, (1) complex*. **[R2011:293]**
208. *Trochanterofemoral joint of walking legs: (0) transverse bicondylar, (1) vertical bicondylar*. **[R2011:296]**
209. *Patellotibial joint of walking legs: (0) dorsal monocondylar, (1) simple bicondylar, (2) vertical bicondylar, dorsal hinge*. **[R2011:300]**
210. *Walking leg femur: (0) shorter than, or equal in length to patella, (1) longer than patella*.
211. *Femoropatellar joint: (0) transverse dorsal hinge, (1) bicondylar articulation, (2) hinge*. **[R2011:301; S2007:69]**
212. *Origin of posterior transpatellar muscle: (0) arises on distodorsal surface of femur, transverses femoropatellar joint ventral to axis of rotation, receives fibres from wall of patella, (1) arises on distal process of femur, transverses femoropatellar joint dorsal to axis of rotation, does not receive fibres from patella*. **[R2011:302]**
213. *Tibiotarsus: (0) separate tibia and tarsus, (1) unjointed tibiotarsus*. **[R2011:303]**
214. *Elastic arthrodial sclerites spanning the tibia-tarsus joints: (0) absent, (1) present*. **[R2011:304]**
215. *Divided tarsus (basitarsus and telotarsus): (0) absent, (1) present*. **[S2007:82]**
216. *Telotarsus in adults with two or more tarsomeres: (0) absent, (1) present*. **[S2007:84]**
217. *Three telotarsomeres on appendages of postoral somites IV-VI: (0) absent, (1) present*. **[S2007:85]**
218. *Tarsal organ: (0) absent, (1) present*. **[R2011:306]**

219. *Pretarsal depressor muscle origin: (0) on tarsus, (1) on tibia or patella*. **[R2011:307]**
220. *Apotele of walking leg I: (0) absent, (1) present*. **[S2007:90]**
221. *Pulvillus: (0) absent, (1) present*. **[S2007:92]**
222. *Exopod of post-mandibular limb multisegmented with inward pointing setae: (0) absent, (1) present*.**[R2012:15]**
223. Distal-less *expression in mandible (or positionally equivalent limb): (0) present (including transient expression in embryo and in palp), (1) absent*. **[R2011:167]**
224. *Mandible (gnathobasic appendage of third limb-bearing metamere is main feeding limb of adult head): (0) absent, (1) basipodite with elaboration of proximal endite, (2) mandible a coxal endite embedded between the labrum and hypopharynx to form a chewing chamber*. **[R2011:168]**
225. *Mandibular base plate forming side of head: (0) absent, (1) present*. **[R2011:169]**
226. *Telognathic mandible with musculated gnathal lobe, flexor arising dorsally on cranium: (0) absent, (1) present*. **[R2011:170]**
227. *Pectinate lamellae on mandible: (0) absent, (1) present*. **[R2011:171]**
228. *Mandibular gnathal edge: (0) consisting of molar and incisor process, (1) only ellipsoid molar present, (2) number of teeth arranged in a row, (3) shovel with terminal teeth, (4) group of paired teeth and hair pad*. **[R2011:172]**
229. *Second (anterior) mandibular cranial articulation: (0) absent, (1) present*. **[R2011:173]**
230. *Ball-and-socket mandibular articulation: (0) absent, (1) present, formed between clypeal condyle and mandibular ridges*. **[R2011:174]**
231. *Mandibular scutes: (0) absent, (1) present*. **[R2011:175]**
232. *Mandibular palp: (0) present, (1) absent*. **[R2011:176]**
233. *Hand-shaped 'movable appendage" between* pars incisiva *and* par molaris *of mandible: (0) absent, (1) present*. **[R2011:177]**
234. *Posterior tentorial apodeme: (0) absent, (1) present as metatentorium, (2) paired separate rods*. **[R2011:178]**
235. *Pre- and metatentorium fused: (0) absent, (1) present*. **[R2011:179]**
236. *Anterior tentorial arms: (0) absent, (1) cuticular tentorium developed as ectodermal invagination, (2) cuticular fulcro-tentorium*. **[R2011:180]**
237. *Posterior suspension of anterior apodemes to cranial wall: (0) absent, (1) present*. **[R2011:181]**
238. *Anterior tentorium: (0) separate, rod-like anterior tentorial apodemes, (1) anterior part of tentorial apodeme forms arched, hollow plate that approach each other mesially but remain separate, (2) anterior tentorium an unpaired roof*. **[R2011:182]**
239. *Swinging tentorium, mandible abducts by tentorial movements: (0) absent, (1) present*. **[R2011:183]**

240. *Mandibular articulation with tentorium: (0) gnathal lobe articulates with epipharyngeal bar, (1) mandible articulates with hypopharyngeal bar*. **[R2011:184]**
241. *Suspensory bar from mandible: (0) absent, (1) present*. **[R2011:185]**
242. *Intergnathal connective lamina: (0) absent, (1) present*. **[R2011:186]**
243. *Mandibulo-hyopharyngeal muscle: (0) absent, (1) present*. **[R2011:187]**
244. *Maxilla 1: (0) absent (locomotory limb on fourth metamere), (1) present*. **[R2011:193]**
245. *First maxillary precoxa: (0) absent, (1) present*. **[R2011:194]**
246. *Number of medially directed lobate endites on first maxilla: (0) two endites, (1) one endite*. **[R2011:195]**
247. *First maxillary palps: (0) present (including telopodite of positionally equivalent limb in chelicerates), (1) absent*. **[R2011:196]**
248. *First maxillary palp hypertrophied: (0) absent, (1) present*. **[R2011:197]**
249. *First maxillary palp an elongate cleaning organ: (0) absent, (1) present*. **[R2011:198]**
250. *First maxilla divided into cardo, stripes, lacinia, and galea. With similar musculature and function: (0) absent, (1) present*. **[R2011:199]**
251. *Interlocking of galea and superligua: (0) absent, (1) present*. **[R2011:200]**
252. *First maxilla coalesced with sterna intermaxillary plate: (0) absent, (1) present, (2) mental elements of gnathochilarium consolidated*. **[R2011:201]**
253. *Second maxillae on fifth metamere: (0) appendage developed as trunk limb, (1) well developed maxilla differentiated as mouthparts, (2) vestigial appendage, (3) appendage lacking, (4) well developed, not a mouth part, (5) long antenniform appendage.* **[R2011:202]**
254. *Egg tooth on second maxilla: (0) absent (no embryonic egg tooth on cuticle of fifth limb-bearing metamere), (1) present*. **[R2011:203]**
255. *Maxillary plate: (0) absent, (1) present*. **[R2011:204]**
256. *Coxae of second maxillae medially fused: (0) absent (coxae of appendages of fifth metamere not fused), (1) present*. **[R2011:205]**
257. *Symphylan-type labium: (0) absent, (1) present*. **[R2011:206]**
258. *Linea ventralis: (0) absent, (1) present*. **[R2011:207]**
259. *Divided glossae and paraglossae: (0) undivided pair of glossae and paraglossae, (1) glossae and paraglossae bilobed*. **[R2011:208]**
260. *Rotation of labial Anlagen: (0) absent, (1) present*. **[R2011:209]**
261. *Widened apical segment of labial palp: (0) absent, (1) present*. **[R2011:210]**
262. *Direct articulation between first and fourth articles of telopodite and maxillipede: (0) absent (first and fourth articles of telopodite of sixth metamere lack a common hinge), (1) present*. **[R2011:212]**
263. *Coxosternite of maxillipede sclerotised in midline: (0) coxae separated medially, with sternite present in adult, (1) coxosternal plates meeting, with flexible hinge, (2) coxosternal plate meeting medially, hinge sclerotised and non-functional*. **[R2011:213]**

264. *Maxillipede coxosternite deeply embedded into cuticle above second trunk segment: (0) not embedded, (1) embedded*. **[R2011:214]**
265. *Maxillipede segment with pleurite forming a girdle around coxosternite: (0) small lateral pleurite, (1) large girdling pleurite*. **[R2011:215]**
266. *Sternal muscles truncated in maxillipede segment, not extending into head: (0) sternal muscles extended into head, (1) sternal muscles truncated*. **[R2011:216]**
267. *Maxillipede tooth plate (anteriorly-projected, serrate coxal endite): (0) absent, (1) present*. **[R2011:217]**
268. *Maxillipede venom gland: (0) absent, (1) present*. **[R2011:218]**
269. *Maxillipede distal segments fused as a tarsungulum: (0) separate tarsus and pretarsus, (1) tarsus and pretarsus fused as tarsungulum*. **[R2011:219]**
270. *Claspers as modified anterior thoracopods (applicable for taxa with phyllopodous limbs only): (0) absent, (1) one pair of claspers (at least movable finger), (2) two pairs of claspers*. **[R2011:251]**
271. *Moveable endite on walking leg coxae: (0) absent, (1) present*. **[S2007:53]**
272. *Length of podomeres VI-4 and VI-5: (0) VI-5 > VI-4, (1) VI-5 = VI-4, (2) VI-4 >VI-5*. **[T2007:17]**
273. *Walking legs II-IV spinose: (0) absent, (1) present*. Characters 273-279 refer to the walking legs of taxa with six pairs of cephalic appendages, i.e. chelicerates, and were first used by ref. 24 (ch. 273-279)
274. *Walking legs V spinose: (0) absent, (1) present*. This character is coded as applicable to eurypterids only. **[L2012:274]**
275. *Leg VI modified into a "pusher": (0) absent, (1) present*. The posteriormost walking legs of xiphosurans possess spatulate tarsal processes, referred to as a 'pusher'. **[L2012:275]**
276. *Leg VI modified into a swimming paddle: (0) absent, (1) present*.
277. *Podomere 9 on appendage VI: (0) absent, (1) present*.
278. *Podomere 7a: (0) absent, (1) present*.
279. *Walking legs chelate: (0) absent, (1) present*.
280. *Endopod segmentation in locomotory limbs: (0) ca. 20 segments, without terminal claw, (1) eight segments (excluding protopodite) and terminal claw, (2) at most five or six segments and terminal (pretarsal) claw.* **[R2011:282]**
281. *Appendage of first thoracic somite underneath the cephalo-thoracic articulation: (0) absent, (1) present*. **[O2013:8]**
282. *Trunk endopods: (0) absent, (1) present*. **[O2013:9]**
283. *Appendage on first opisthosomal segment: (0) appendage present in post-embryonic stage, (1) appendage absent.* **[R2011:247]**
284. *Appendage of seventh metamere incorporated into cephalic tagmata: (0) absent, (1) present*. The anterior trunk appendages of xiphosurans are incorporated into the cephalon and are functionally part of the head. **[L2012:284]**
285. *Appendage VII greatly reduced and plate-like: (0) absent, (1) present*.
286. *Limb VII as chilaria: (0) absent, (1) present*. **[R2011:248]**

287. *Pectines: (0) absent, (1) present*. **[R2011:249]**
288. *Plate-like opisthosomal appendages: (0) absent, (1) present*. In euchelicerates the mesosomal appendages typically lack endopods and are modified into plate-like operculae. **[L2012:288]**
289. *Opisthosomal silk glands and spigots: (0) absent, (1) present*.
290. *Opisthosomal spinnerets: (0) absent, (1) present*.
291. *Opisthosomal spinnerets, locations: (0) near middle of opisthosoma, (1) near posterior of opisthosoma*. **[S2007:113]**
292. *Anterior medial 'spinnerets': (0) absent, (1) present*. **[S2007:114]**
293. *Transverse sutures on ventral plate: (0) absent, (1) present*. The mesosomal opercula are fused into a single unit in some eurypterids and arachnids. In chasmataspidids and some eurypterids lateral operculae are separated by a transverse suture. **[L2012:293]**
294. *Anterior opercula plate: (0) absent, (1) present*. **[L2012:294**
295. *Meso- and metathorax in mature stage bearing wings: (0) absent, (1) present*. **[R2011:255]**
296. *Wing flexion: (0) absent, (1) present*. **[R2011:256]**
297. *Furcula: (0) absent, (1) present*. **[R2011:291]**
298. *Coxal vesicles: (0) absent, (1) present, (2) eversible*. **[R2011:289]**
299. *Styli: (0) absent, (1) present*. **[R2011:290]**
300. *Trunk limbs with lobate endites formed by folds in limb bud: (0) absent, (1) present*. **[R2011:281]**
301. *Trunk endopod endites: (0) spiniferous, (1) rounded.* **[R2012:13]**
302. *Spiniferous endites differentiated into primary (small) and secondary (long) sets: (0) absent, (1) present*.
303. *Trochanteronotal muscle: (0) absent, (1) present*. **[R2011:294]**
304. *Trochanter distal joint: (0) mobile, (1) short, ring-like trochanter lacking mobility at joint with prefemur*. **[R2011:295]**
305. *Unique trochanteral femur-twisting muscle: (0) absent, (1) present*. **[R2011:297]**
306. *Unique femur-tibia pivot joint: (0) absent, (1) present*. **[R2011:298]**
307. *Patella/tibia joint: (0) free, (1) fused*. **[R2011:299]**
308. *Tarsus segmentation (in hexapods): (0) not subsegmented, (1) subsegmented*. **[R2011:305]**
309. *Pretarsal levator muscle: (0) present, (1) absent (depressor is sole pretarsal muscle)*. **[R2011:308]**
310. *Pretarsal claws: (0) paired, (1) unpaired*. **[R2011:309]**
311. *Pretarsal claw articulation: (0) on pretarsal base, (1) on distal tarsomere*. **{R2011:310]**
312. *Plantulae: (0) absent, (1) present*. **[R2011:311]**
313. *Trunk exopod setae: (0) short, fine setae, (1) long, flattened, tapering distally, with slight separation, (2) lamellate setae, (3) widely space filamentous setae*. **[***cf.* **R2011:242;** *cf*. **E2011:29]**
314. *Paddle-like epipods: (0) absent, (1) present*. **[R2011:286]**

315. *Cilia: (0) present in several organ systems, including photoreceptor, nephridia, and genital tracts, (1) present in (at most) sperm*. **[R2011:22]**
316. *Bismuth staining of Golgi complex beads: (0) absent, (1) present*. **[R2011:28]**
317. *Circumoesophageal brain: (0) absent, (1) present*.
318. *Immigration of cells during neurogenesis: (0) segregation and immigration of single cells, (1) immigrating clusters of post-mitotic cells*. **[R2011:47]**
319. *Segmental invaginations of neuroectoderm giving rise to ventral organs: (0) absent, (1) present*. **[R2011:48]**
320. *Neuronal stem cells with asymmetrical division: (0) absent, (1) present*. **[R2011:49]**
321. *Cellular derivatives of neuroectoderm: (0) neuroectoderm generates epidermal and neural cells, (1) central area of neuroectoderm generates neural cells only*. **[R2011:50]**
322. *Early differentiating neurons aCC, pCC, RP2, U-CQ, EL and AUN: (0) absent, (1) present*. **[R2011:51]**
323. *EC neurons: (0) Eca and Ecp only,(1) Eca, Ecp and EcI*. **[R2011:52]**
324. *Anterior pair of serotonergic neurons with neurites that cross to contralateral side: (0) absent, (1) present*. **[R2011:53]**
325. *Posterior pair of serotonergic neurons with neuritis that cross to contralateral side: (0) absent, (1) present*. **[R2011:54]**
326. *Serotonergic somata clustered: (0) unclustered, (1) clusters of about 10 cells*. **[R2011:55]**
327. *Serotonergic cell group ‘b’ of* Harzsch (2004)*: (0) absent, (1) present*. **[R2011:56]**
328. *Single median serotonergic neurons ‘c’ and ‘d’ of* Harzsch (2004)*: (0) absent, (1) present*. **[R2011:57]**
329. *Globuli cells: (0) confined mainly to brain, in massive clusters, (1) making up majority of neuropil and ventral layer of ventral nerve cord*. **[R2011:58]**
330. *Corpora allata: (0) absent, (1) present*. **[R2011:59]**
331. *Intrinsic secretory cells in protocerebral neurohemal organ: (0) absent, (1) present*. **[R2011:60]**
332. *Enlarged epipharyngeal ganglia: (0) absent, (1) present*. **[R2011:61]**
333. *Innervation of mouth area by anterior stomogastric nervous system: (0) absent, (1) present*. **[R2011:62]**
334. *Ganglia of pre-oesophageal brain: (0) protocerebrum, (1) protocerebrum and deutocerebrum, (2) proto-, deuto- and tritocerebra*. **[R2011:63]**
335. *Ganglia of post-oral appendages fused into single nerve mass: (0) absent, (1) present*. **[R2011:64]**
336. *Bilateral neuropils: (0) absent, (1) present*. **[R2011:65]**
337. *Fan-shaped body in brain: (0) absent, (1) present*. **[R2011:66]**
338. *Midline neuropil (m11): (0) absent, (1) present*. **[R2011:67]**
339. *Midline neuropil 2: (0) absent, (1) present*. **[R2011:68]**
340. *Position of midline neuropil: (0) superficial to protocerebrum, (1) embedded within protocerebral matrix*. **[R2011:69]**

341. *Looped axons provide local tangential connections to midline neuropil: (0) absent, (1) present*. **[R2011:70]**
342. *Neurons defining modules to midline neuropil: (0) heterolateral neurons, the cells bodies of which are remote from neuropil, (1) perpendicular local neurons*. **[R2011:71]**
343. *Size variation in somata supplying midline neuropil: (0) uniform, (1) variable*. **[R2011:72]**
344. *Olfactory lobes linked to a lateral component of protocerebrum by olfactory globular tract: (0) absent, (1) present*. **[R2011:73]**
345. *Segmental association of glomeruli: (0) linked to specific appendages / ganglia, (1) supplied by all legs / ganglia*. **[R2011:74]**
346. *Arcuate body in brain: (0) absent, (1) present*. **[R2011:75]**
347. *Ellipsoid body in brain: (0) absent, (1) present*. **[R2011:76]**
348. *Nobuli in brain: (0) absent, (1) present*. **[R2011:77]**
349. *Protocerebral bridge: (0) absent, (1) present*. **[R2011:78]**
350. *Mushroom body calyces: (0) absent, (1) present*. **[R2011:79]**
351. *Deutocerebral olfactory lobe with glomeruli: (0) absent, (1) present*. **[R2011:80]**
352. *Deutocerebral olfactory-globular tract: (0) absent, (1) uncrossed, (2) with chiasma*. **[R2011:81]**
353. *Deutocerebrum with bipartite antennular neuropils: (0) absent, (1) present*. **[R2011:82]**
354. *Rhabdomeric lateral eyes with new elements formed at a proliferation zone at side of developing eye field: (0) absent, (1) present*. **[R2011:94]**
355. *Form of lateral eyes: (0) facetted, (1) simple lens with cup-shaped retina, (2) stemmata*. **[R2011: 95]**
356. *Proliferation zone of lateral eye field: (0) row-by-row addition, (1) intermediate (*Scutigera*) type, (1) morphogenetic front*. **[R2011:96]**
357. *Compound eyes medial margins: (0) separate, (1) medially contiguous, (2) fused*. **[R2011:97]**
358. *Compound eye stalked, basally articulated: (0) absent (eye sessile), (1) present*. **[D2009:12; R2011:98]**
359. *Position of lateral facetted eyes: (0) ventral, (1) dorsal*. **[P2010:3]**
360. *Four or five ventral eyes arranged in a subtransverse band across head shield: (0) absent, (1) present*. **[E2011:9]**
361. *Visual surface with calcified lenses bounded by circumocular suture: (0) absent, (1) present*. **[C2004:21; P2010:4]**
362. *Dorsal bulge in exoskeleton accommodating drop-shaped ventral eyes: (0) absent, (1) present*. **[O2013:22]**
363. *Eye slits: (0) absent, (1) present*. **[O2013:23]**
364. *Compound eyes internalised early in ontogeny, shifted dorsally into a cuticular pocket: (0) absent, (1) present*. **[R2011:99]**
365. *Ophthalmic ridges (0) absent, (1) present*. **[R2011:100]**
366. *Number of corneagenous cells: (0) many, (1) two*. **[R2011:101]**

367. *Corneagenous cells containing pigment grains: (0) corneagenous cells lacking pigment grains, (1) corneagenous cells are primary pigment cells*. **[R2011:102]**
368. *Bipartite distal pigment cells with an inner pigment-free portion, and an outer pigment-bearing portion separated by an extracellular space: (0) distal pigment cells not bipartite, (1) distal pigment cells bipartite*. **[R2011:103]**
369. *Interommatidial pigment cells attached to cornea and basement membrane: (0) absent, (1) present*. **[R2011:104]**
370. *External pigment cells covering whole ocellar corpus: (0) absent, (1) present*. **[R2011:105]**
371. *Ommatidium with crystalline cone: (0) cone absent, (1) cone present*. **[R2011:106]**
372. *Crystalline cone cells: (0) tetrapartite crystalline cone, lacking accessory cells, (1) cone bipartite, with two accessory cells, (2) pentapartite cone, (3) tripartite cone*. **[R2011:107]**
373. *Reduction of processes of crystalline cone-producing cells: (0) all cells have processes that pass through clear zone and rhabdom, (1) only accessory cells have processes*. **[R2011:108]**
374. *Distally displaced nuclei of accessory crystalline cone cells: (0) absent, (1) present*. **[R2011:109]**
375. *Clear zone between dioptric apparatus and retina: (0) absent (apposition eye), (1) present (superposition eye)*. **[R2011:110]**
376. *Circumretinular sheath cells: (0) absent, (1) present*. **[R2011:111]**
377. *Optic chiasma between lamina and medulla: (0) absent, (1) present*. **[R2011:112]**
378. *Medulla divided into two layers by Cuccati bundle: (0) undivided, (1) divided*. **[R2011:113]**
379. *Lobula or protolobula receiving crossed axons from medulla: (0) absent, (1) present*. **[R2011:114]**
380. *Third optic neuropil (lobula) separated from protocerebrum: (0) absent, (1) present*. **[R2011:115]**
381. *Fourth optic neuropil (lobula plate), receiving uncrossed axons from medulla: (0) absent, (1) present*. **[R2011:116]**
382. *Lateral eye rhabdomes with quadratic network: (0) absent, (1) present*. **[R2011:117]**
383. *Number of median eyes: (0) none, (1) two, (2) three, (3) four, (4) one (embryonic), (5) one (potential fused) associated with lateral eyes*. **[R2011:118]**
384. *Inverted median eye: (0) absent, (1) present*. **[R2011:119]**
385. *Median eyes fused to naupliar eyes: (0) absent, (1) present*. **[R2011:120]**
386. *Type of naupliar eye: (0) inverse, (1) everse*. **[R2011:121]**
387. *Naupliar eye shape: (0) globular, (1) triangular*. **[R2011:122]**
388. *Tapetal cells in cups of naupliar eye: (0) absent, (1) present*. **[R2011:123]**

389. *Dorsal frontal organ (malacostracan type): (0) absent, (1) present*. **[R2011:124]**
390. *Ocular tubercle: (0) absent, (1) present*. **[R2011:125]**
391. *Position of ocular tubercle: (0) central, (1) anterior*.
392. *Slit sensilla: (0) absent, (1) present*. **[R2011:46]**
393. *Trichobothria innervated by several sensory cells, with dendrites having only indirect contact with the hair base: (0) absent, (1) present*. **[R2011:126]**
394. *Basal bulb in trichobothria: (0) absent, (1) present*. **[R2011:127]**
395. *Appendage III (arachnid leg 1) with coxapophyses forming preoral chamber: (0) absent, (1) present.* **[S2007:50]**
396. *Shape of coxapophyses 1: (0) triangular, (1) spatulate, (2) lacrimiform*. Scorpions possess elongate coxapophyses on their anterior walking leg coxae (ch. 395). Extant taxa tend to possess spatulate or lacrimiform coxapophyses, whereas Palaeozoic taxa tend to have more triangular coxapophyses. **[L2012:396]**
397. *Appendage IV (arachnid leg 2) with coxapophyses forming preoral chamber: (0) absent, (1) present*. **[S2007:51]**
398. *Post-cephalic filter feeding apparatus with sternitic food grooves: (0) absent, (1) present*. **[R2011:145]**
399. *Salivary gland position: (0) ectodermal, on second maxilla, (1) mesodermal segmental organs on first maxilla*. **[R2011:191]**
400. *Maxillary salivary gland opening: (0) pair of opening at base of second maxilla, (1) median opening at midventral groove of labium, (2) median opening on salivarium, between labium and hypopharynx*. **[R2011:192]**
401. *Mouth position: (0) terminal, (1) ventral*. **[R2011:128]**
402. *Head / mouth orientation: (0) prognathous, mouth directed anteroventrally, (1) hypognathous, mouth directed ventrally, (2) mouth directed posteriorly, (1) mouth directed ventrally (non-hypognathous)*. **[R2011:129]**
403. *Proboscis: (0) absent, (1) present*.
404. *Radially arranged cicumoral structures (papillae, plates or lamellae): (0) absent (bilaterally symmetrical mouthparts), (1) present*. **[R2011:130]**
405. *Circumoral structures as a circlet of overlapping plates with teeth on their inner margins (“peytoia apparatus”): (0) absent, (1) present*. **[R2011:131]**
406. *Epistome-labrum / clypeolabrum: (0) absent, (1) present*. This character is coded as present for taxa possessing a hypostome[40]. **[R2011:132]**
407. *Sclerotization of epistome-labrum / clypeolabrum (hypostome): (0) absent, (1) present*.
408. *Hypostome attachment: (0) wide attachment, with or without suture, (1) natant, (2) narrow overlap with pre-hypostome, (3) narrow attachment at hypostomal suture*. **[P2010:12]**
409. *Hypostome divided into anterior and posterior parts by transverse suture: (0) absent, (1) present*. **[P2010:13]**
410. *Fleshy labrum: (0) absent, (1) present*. **[R2011:133]**

411. *Entognathy (overgrowth of mandibles and maxillae by cranial folds): (0) absent, (1) present*. **[R2011:134]**
412. *Admentum differentiated latero-ventrally on each side of head capsule, developed from posterior part of mouth fold: (0) absent, (1) present*. **[R2011:135]**
413. *Sclerotic sternum formed by antennal to maxillulary sternites, including paragnaths on mandibular sternum: (0) absent, (1) present*. **[R2011:136]**
414. *Intercheliceral epipharygeal sclerite: (0) absent, (1) present*. **[S2007:191]**
415. *Tritosternum: (0) absent, (1) present*. **[R2011:137]**
416. *Postcerebral pharynx: (0) absent, (1) present*. **[S2007:199]**
417. *Hypopharynx: (0) absent or only median lingua, (1) complete hypopharynx of lingua and paired superlinguae*. **[R2011:138]**
418. *Fulturae: (0) absent or limited to a hypopharyngeal suspensor, (1) present, in a groove between arthrodial membrane of maxilla and labium*. **[R2011:139]**
419. *Posterior process of tentorium fused anteriorly with hypopharyngeal bar and transverse bar: (0) absent, (1) present*. **[R2011:140]**
420. *Triradiate pharyngeal lumen: (0) absent, (1) present*. **[R2011:141]**
421. *Flexible buccal tube and stylet apparatus: (0) absent, (1) present*. **[R2011:142]**
422. *Three-branched epistomal skeleton supporting the pharyngeal dilator muscles: (0) absent, (1) present*. **[R2011:143]**
423. *U-shaped anterior gut diverticulae: (0) absent, (1) present*.
424. *Proventriculus (in the foregut): (0) absent, (1) present*. **[R2011:228]**
425. *Lateralia and inferolateralia anteriores in the cardiac chamber: (0) absent, (1) present*. **[R2011:229]**
426. *Unpaired superomedianum at transition from cardia to pyloric chamber: (0) absent, (1) present*. **[R2011:230]**
427. *Inferomedianum anterius (midventral cardiac ridge): (0) absent, (1) present*. **[R2011:231]**
428. *Inferomedianum posterius (midventral pyloric ridge): (0) absent, (1) present*. **[R2011:232]**
429. *Atrium between inferomediana connecting cardiac primary filter grooves with pyloric filter grooves: (0) absent, (1) present*. **[R2011:233]**
430. *Gut caecae: (0) absent, (1) present along the midgut, (2) restricted to the anterior part of the midgut*. **[R2011:234]**
431. *Serially repeating midgut glands: (0) absent, (1) reniform, metameric midgut glands with submillimetric lamellae, (2) radiating tubular diverticula.* **[D2009:16; R2011:235]**
432. *Proctodeal dilation: (0) posterior section of hindgut simple, lacking a dilation, (1) proctodeum having a rectal ampulla with differentiated papillae*. **[R2011:236]**
433. *Pyloric region with ring of flattened cells with thick intima: (0) absent, (1) present*. **[R2011:237]**
434. *Peritrophic membrane: (0) absent, (1) present*. **[R2011:238]**

435. *Tubular, radiating diverticula with intracellular digestion: (0) absent, (1) present*. **[R2011:239]**
436. *Metanephridia with sacculus with podocytes: (0) absent, (1) present*. **[R2011:29]**
437. *Distribution of segmental glands: (0) on many segments, (1) in at most last four segments and first two post-cephalic segments, (2) on second antennal and maxillary segments only*. **[R2011:30]**
438. *Maxillary nephridia: (0) absent in postembryonic stadia, (1) paired, (2) fused nephridia of both maxillary segments*. **[R2011:31]**
439. *Coxal gland orifice, leg I: (0) absent, (1) present*. **[R2011:32]**
440. *Tömösváry organ ("temporal organs" at side of head behind insertion of antennules): (0) absent, (1) present*. **[R2011:33]**
441. *Salivary gland reservoir: (0) absent, (1) present*. **[R2011:34]**
442. *Malpighian tubules formed as endodermal extensions of the midgut: (0) absent, (1) present*. **[R2011:35]**
443. *Malpighian tubules formed as ectodermal extensions of the hindgut: (0) absent, (1) single pair of juncture of midgut and hindgut, (2) multiple pairs at anterior end of hindgut*. **[R2011:36]**
444. *Form of ectodermal Malpighian tubules: (0) elongate, (1) papillate*. **[R2011:37]**
445. *Neck organ: (0) absent, (1) present*. **[R2011:38]**
446. *Coxal organs on last pair of legs: (0) absent, (1) present*. **[R2011:378]**
447. *Pair of repugnatorial glands in the carapace: (0) absent, (1) present*. **[R2011:380]**
448. *Pleural defense glands with benzoquinones: (0) absent, (1) present*. **[R2011:381]**
449. *Aculeus with sting / opisthosomal venom gland: (0) absent, (1) present*. **[R2011:382]**
450. *Shape of aculeus: (0) rounded, (1) lacrimiform*.
451. *Posterior oblique muscles of Box-Truss Axial Muscle System (BTAMS): (0) absent, (1) present*. **[2007:127]**
452. *Endosternum (ventral tendons fused into prosomal endosternum): (0) absent, (1) present*. **[R2011:259]**
453. *Endosternum fenestrate: (0) absent, (1) present*. **[S2007:130]**
454. *Suboral suspensor: (0) absent, (1) present*. **[S2007:131]**
455. *Ventral endosternal suspensor attached on coxa of anterior adjacent somite: (0) absent, (1) present*. **[S2007:133]**
456. *Dorsal endosternal suspensor of fourth postoral segment with anterolateral carapacal insertion: (0) absent, (1) present*. **[R2011:260]**
457. *Trunk endoskeleton in each segment: (0) pair of lateral connective plates, (1) pair of sternocoxal rods (ventral apodemes), (2) complex connective endosternite, (3) mainly cuticular, composed of two intrasegmental furcal arms and intersegmental spinal process*. **[R2011:269]**
458. *Oblique muscle layer in body wall: (0) no oblique layers, (1) oblique layers*. **[R2011:220]**

459. *Longitudinal muscles attach to intersegmental tendons: (0) absent, (1) present*. **[R2011:275]**
460. *Longitudinal muscles: (0) united sternal and lateral longitudinal muscles, (1) separate sternal and longitudinal muscles, with separate segmental tendons*. **[R2011:221]**
461. *Superficial pleural muscles: (0) absent, (1) present*. **[R2011:222]**
462. *Crossed, oblique dorsoventral muscles: (0) absent, (1) present*. **[R2011:223]**
463. *Deep dorsoventral muscles in the trunk: (0) absent, (1) present*. **[R2011:224]**
464. *Circular body muscle: (0) present, (1) present*. **[R2011:225]**
465. *Discrete segmental cross-striated muscles attached to cuticular apodemes: (0) absent, (1) present*. **[R2011:226]**
466. *Trunk muscles: (0) straight, (1) twisted*. **[R2011:227]**
467. *Haemoglobin: (0) absent, (1) present*. **[R2011:39]**
468. *Subcutaneous hemal channels in body wall: (0) absent, (1) present*. **[R2011:40]**
469. *Dorsal heart with segmental ostia and pericardial sinus: (0) absent, (1) present*. **[R2011:41]**
470. *Internal valves formed by lips of ostiae projecting deeply into heart lumen (to prevent haemolymph backflow within the heart): (0) absent, (1) present.* **[R2011:42]**
471. *Circumesophageal circulatory loop with ventral trumpet-shaped opening toward head: (0) absent, (1) present*. **[R2011:43]**
472. *Aorta descendens connects heart and ventral vessel: (0) absent, (1) present*. **[R2011:44]**
473. *Myoarterial formation with aortic dilations associated with oesophageal dilator muscles: (0) absent, (1) present*. **[R2011:45]**
474. *Respiratory exites: (0) absent, (1) present*.
475. *Dorsal bands of blade-like gills: (0) absent, (1) present*. **[D2009:41; R2011:261]**
476. *Lamellate respiratory organ derived from posterior wall of opisthosomal limb buds: (0) absent, (1) present*. **[R2011:243]**
477. *Spines on lamellar margins: (0) absent, (1) present*.
478. *Shape of pillars of haemolymph space inside gill/lung lamellae: (0) at least two perikarya meeting midway in the haemolymph space, (1) pillars, including a strong axis of microtubules*.
479. *Lamellar orientation: (0) horizontal, (1) vertical*.
480. *Respiratory lamellae on opisthosomal somite 2: (0) absent, (1) present*. **[S2007:121]**
481. *Respiratory lamellae on opisthosomal somite 3: (0) absent, (1) present*. **[S2007:122]**
482. *Respiratory lamellae on opisthosomal somites 4-6: (0) absent, (1) present*. **[S2007:123]**
483. *Respiratory lamellae on opisthosomal somites 7: (0) absent, (1) present*. **[S2007:124]**

484. *Tracheae / spiracles: (0) absent, (1) pleural spiracles, (2) spiracles at bases of walking legs, opening into tracheal pouches, (3) single pair of spiracles on head, (4) dorsal spiracle opening to tracheal lungs, (5) open-ended tracheae with spiracle on second opisthosomal segment, (6) many spiracles scattered on body, (7) pair of spiracles in the collar region, (8) four pairs of opisthosomal stigmata with irregular unprotected openings, (9) opening on opisthosomal somites 3 and 4*. **[R2011:312]**
485. *Longitudinal and transverse connections between segmental tracheal branches: (0) tracheae not connected, (1) tracheae connected*. **[R2011:313]**
486. *Pericardial tracheal system with chiasmata: (0) dendritic tracheae, (1) long, regular pipe-like tracheae with specialised moulting rings*. **[R2011:314]**
487. *Abdominal spiracles: (0) present (pleural spiracles on posterior part of trunk), (1) absent on first abdominal segment, (2) absent on all abdominal segment*. **[R2011:315]**
488. *Intrasternite stigmata: (0) absent (opening to book-lungs at margin of sternite), (1) present*. **[R2011:246]**
489. *Crural glands: (0) absent, (1) present*. **[R2011:379]**
490. *Female abdomen with ovipositor formed by gonapophyses of segments VIII and IX: (0) absent, (1) present*. **[R2011:331]**
491. *Gonangulum sclerite fully developed as ovipositor base, articulating with tergum IX and attached to 1st valvula / valvifer: (0) absent, (1) present*. **[R2011:332]**
492. *Gonads: (0) primarily prosomal, (1) primarily opisthosomal*. **[S2007:151]**
493. *Ladder-like opisthosomal glands: (0) absent, (1) present*. **[S2007:152]**
494. *Male gonads on two distinct parts, one producing sperm and another producing a holocrine secretion similar to degenerate sperm: (0) absent, (1) present*. **[S2007:153]**
495. *Genital opening appearing to open in prosomal region: (0) absent, (1) present*. **[S2007:155]**
496. *Ovipositor: (0) absent, (1) present*. **[R2011:333]**
497. *Sclerotised spermatophore: (0) absent, (1) present*.
498. *Stalked spermatophore attached to substratum: (0) absent, (1) present*.
499. *Legs of seventh trunk segment transformed into gonopods: (0) absent, (1) present*. **[R2011:334]**
500. *Dignathan-type penes: (0) absent, (1) present*. **[R2011:335]**
501. *Penis (spermatopositor) opening on anteroventral part of opisthosoma: (0) absent, (1) present*. **[R2011:336]**
502. *Penis form: (0) short, membranous, undivided, (1) long, chitinous, divided into shaft and glans*. **[R2011:337]**
503. *Genital appendage: (0) absent, (1) present*.
504. *Male parameres: (0) undifferentiated, (1) pair of lateral plates on segment XI, (2) pair of parameres on segment IX, (3) incorporated into phallic apparatus as sclerite*. **[R2011:338]**
505. *Penis on abdominal segment IX: (0) absent, (1) present*. **[R2011:339]**

506. *Female gonopore position: (0) on same somite as male, (1) two segments anterior to male, (2) six segments anterior to male, (3) seven segments anterior to male*. **[R2011:340]**
507. *Female gonopore parity: (0) paired, (1) medium, unpaired*. **[R2011:341]**
508. *Genital operculum divided, incorporated into pedicel: (0) absent, (1) present*. **[R2011:342]**
509. *Lacrimiform genital operculae: (0) absent, (1) present*.
510. *Genital operculum overlapping third opisthosomal sternite: (0) absent, (1) present*. **[R2011:343]**
511. *Postgenital appendages: (0) opercular and/or lamellar, (1) poorly sclerotized or eversible, (2) absent*. **[R2011:344]**
512. *Embryonic gonoduct origin: (0) gonoduct arising as a mesodermal coelomoduct, (1) gonoduct arising as a secondary ectodermal ingrowth, (2) gonoduct arising in association with splanchnic mesoderm*. **[R2011:345]**
513. *Lateral testicular vesicles linked by central, posteriorly-extended deferens duct: (0) absent, (1) present*. **[R2011:346]**
514. *Testiclar follicles with pectinate arrangement: (0) absent (elongate testicular sac or sacs), (1) several pectinate follicles present*. **[R2011:347]**
515. *Spermatophore web produced by 'Spingriffel' structure: (0) absent, (1) present*. **[R2011:348]**
516 *Sperm dimorphism (microsperm and macrosperm): (0) absent, (1) present*. **[R2011:350]**
517. *Acrosomal complex in sperm: (0) bilayered (filamentous actin perforatorium present), (1) monolayered (perforatorium absent), (2) acrosome absent*. **[R2011:351]**
518. *Pseudoacrosome with dorsal ribbon, granulosome, apical membrane and pseudoacrosomal granular material: (0) absent, (1) present*. **[R2011:352]**
519. *Perforatorium bypasses nucleus: (0) absent (perforatorium penetrates nucleus), (1) present*. **[R2011:353]**
520. *Periacrosomal material: (0) absent, (1) present*. **[R2011:354]**
521. *Striated core in subacrosomal space: (0) absent, (1) present*. **[R2011:355]**
522. *Centrioles in sperm: (0) proximal and distal centrioles present, perpendicular to each other, (1) coaxial centrioles, (2) single centriole, (3) centrioles absent, (4) doublet centrioles with radial 'foot'*. **[R2011:356]**
523. *Centriole adjunct: (0) absent, (1) present*. **[R2011:357]**
524. *Sperm 'accessory bodies' developed from the centriole: (0) absent, (1) present*. **[R2011:358]**
525. *Cristate, non-crystalline mitochondrial derivatives in sperm: (0) absent, (1) present*. **[R2011:359]**
526. *Three filamentous mitochondria symmetrically derivatives in sperm: (0) absent, (1) present*. **[R2011:360]**
527. *Connecting bands between axoneme and mitochondria: (0) absent, (1) present*. **[R2011:361]**

528. *Axoneme parallels entire length of nucleus: (0) absent, (1) present*. **[R2011:362]**
529. *Supernumary axonemal tubules (peripheral singlets): (0) absent, (1) present, formed from the manchette, (2) present, formed from axonemal doublets*. **[R2011:363]**
530. *Number of protofilaments in wall of accessory tubules: (0) 13, (1) 16*. **[R2011:364]**
531. *Mediodorsal peripheral doublet (doublet 1) connected to dorsal ribbon by an obliquely orientated membrane: (0) absent, (1) present*. **[R2011:365]**
532. *Axonemal endpiece 'plume': (0) endpiece not extended, (1) endpiece extended, plume-like*. **[R2011:366]**
533. *Sperm motility: (0) present, (1) absent*. **[R2011:367]**
534. *Nucleus of sperm forms spiral ridge: (0) absent, (1) present*. **[R2011:368]**
535. *Sperm nucleus with manchette of microtubules: (0) absent, (1) present*. **[R2011:369]**
536. *Coiling of spermatozoa flagellum: (0) absent (filiform), (1) present*. **[R2011:370]**
537. *Medial microtubules in spermatozoan axoneme: (0) 9+2, (1) 9+3, (2) 9+0, (3) 12+0, (4) 9+1*. [**R2011:371]**
538. *Sperm conjugation: (0) absent, (1) present*. **[R2011:372]**
539. *Female spermathecae formed by paired lateral pockets in mouth cavity: (0) absent, (1) present*. **[R2011:373]**
540. *Ovary shape: (0) sac- or tube shaped, entire, (1) divided into ovarioles, (2) ovarian network*. **[R2011:374]**
541. *Location of ovary germarium: (0) germarium forms elongate zone in the ventral or lateral ovarian wall, (1) germarium in the terminal part of each egg tube, (2) single, median mound-shaped germarium on the ovarian floor, (3) paired germ zones on ovarian wall, (4) median zone on ovarian roof*. **[R2011:375]**
542. *Site of oocyte growth: (0) in ovarian lumen, (1) on outer surface of ovary, in haemocoel, connected by egg stalk*. **[R2011:376]**
543. *Ventral marsupium formed by oostegites: (0) absent, (1) present*. **[R2011:377]**

Development

544. *Non-migratory gastrulation (extremely limited movement of small-celled presumptive areas: (0) absent, (1) present.* **[R2011:1]**
545. *Early cleavage: (0) total cleavage with radially oriented position of cleavage products, (1) intralecithal cleavage (nuclei undergo several divisions within the yolk mass without concurrent cytokinesis)*. **[R2011:2]**
546. *Role of serosa (= cuticle secreting membrane) in definitive dorsal closure (formation of dorsal body wall): (0) serosa and embryo contribute to definitive*

dorsal closure, (1) provisional dorsal closure (serosa degenerates without contributing to definitive dorsal closure). **[R2011:3]**

547. *Differentiation of amnion as provisional dorsal closure of embryo: (0) absent, (1) present*. **[R2011:4]**
548. *Blastokinesis: (0) absent, (1) open amnionic cavity, (2) closed amnionic cavity, amnioserosal fold fuses beneath the embryo*. **[R2011:5]**
549. *Blastodermal cuticle (cuticular egg envelope): (0) absent, (1) present*. **[R2011:6]**
550. *Ectoteloblasts (forming part of metanaupliar/egg-naupliar region of germ band): (0) absent, (1) present at anterior border of blastopore*. **[R2011:7]**
551. *Caudal papilla: (0) absent, (1) present, anteroventrally folded, derived from preanal growth zone*. **[R2011:8]**
552. *Origin of fat body: (0) fat body cells developed from vitellophages in yolk, (1) fat body cells developed from walls of mesodermal somites*. **[R2011:9]**
553. *Midgut developed into yolk: (0) midgut cells enclose the yolk, (1) lumen of embryonic midgut lacking yolk globules*. **[R2011:10]**
554. *Fate map ordering of embryonic tissues: (0) presumptive mesoderm posterior to presumptive midgut, (1) presumptive mesoderm anterior to midgut, (2) mesoderm midventral, cells sink and proliferate, midgut internalises during cleavage, (3) mesoderm diffuse through ectoderm, (4) midgut develops from anterior/posterior rudiments at each end of midventral mesoderm band*. **[R2011:11]**
555. *Fate of embryologic growth zone (in chelicerates): (0) with a growth zone rising to both the prosoma and opisthosoma, (1) with a growth zone giving rise to the opisthosoma*. **[R2011:12]**
556. *Epimorphic development: (0) absent, (1) present*. **[R2011:13]**
557. *Nauplius larva (orthonauplius) or egg-nauplius: (0) absent, (1) present*. **[R2011:14]**
558. *Pupoid stage (motionless stage after hatching, pupoid remains encased in embryonic cuticle): (0) absent, (1) present*. **[R2011:15]**
559. *Imaginal (pre-adult) moult: (0) present, (1) absent*. **[R2011:16]**
560. *Cyclic parthenogenesis as part of the life cycle: (0) absent, (1) present*. **[R2011:17]**

Behaviour

561. *Tail fan escape reaction: (0) absent, (1) present*. **[R2011:322]**
562. *Egg cluster guarded until hatching, female coiling around egg cluster: (0) absent, (1) female coils ventrally around cluster, (2) female coils dorsally around egg cluster*. **[R2011:328]**
563. *Peripatoid and foetoid stages protected by mother: (0) absent, (1) present*. **[R2011:329]**

564. *Female gonopod used to manipulate single egg: (0) absent, (1) present*. **[R2011:330]**
565. *"By-passing" foreplay, spermatophore transfer on web, "waiting" ritual by females: (0) absent, (1) present*. **[R2011:349]**

Gene order and gene expression

566. Labial *expression domain: (0) expressed over multiple segments, (1) expression confined to second antenna/intercalary segment*. **[R2011:383]**
567. Proboscipedia *expression domain: (0) collinear with labial and deformed domains, (1) anterior boundary of main expression domain of* proboscipedia *behind anterior boundary of* Deformed. **[R2011:384]**
568. Deformed *expression domain: (0) expressed over three or more segments, (1) expression confined to mandibular and first maxillary segment*. **[R2011:385]**
569. Antennipedia *expression domain: (0) strong throughout trunk, (1) restricted from the posterior of the embryo*. **[R2011:386]**
570. *Antisense* Ubx *expression: (0) absent, (1) present.*
571. *Relative position of COI and COII: (0) COI/COII, (1) COI/L2/COII*. **[R2011:387]**
572. *Relative position of tRNA-L: (0) 1su rRNA/L1/L2/NADH 1, (1) 1su rRNA/L1/NADH 1, (2) NADH 1/H'/1su rRNA/L1, (3) 1su rRNA/NADH 1, (4) 1su rRNA/L1/L2/Cytb*. **[R2011:388]**
573. *Relative postion of tRNA-R and tRNA-N: (0) R/N, (1) R/K/N, (2) N/E/R, (3) N/A/S1/R, (4) R/S1*. **[R2011:389]**
574. *Relative position of tRNA-C and tRNA-Y: (0) Y/C, (1) Y/Q/C, (2) C/Q, (3) Q/Y/C, (4) Q/Y/F, (5) Q/I/C*. **[R2011:390]**
575. *Relative position of tRNA-P and tRNA-T: (0) T/-P, (1) –P/T, (2) T between W and cytb, (3) P between nad4L and nad1, (4) T between S2 and nad1*. **[R2011:391]**
576. Distal-less *expression domain in limbs: (0) expression throughout entire medial and distal part of appendage, (1) 'ring and sock' pattern (gap in expression between tibia and femur)*. **[R2011:392]**
577. Dachshund *expression domain in limbs: (0) expression proximally (in trochanter and femur), (1) expression distally in a narrow band*. **[R2011:393]**
578. Extradenticle *expression domain in limbs: (0) expression present in distal part of leg, (1) expression confined to proximal part of leg*. **[R2011:394]**
579. Homothorax *expression domain in limbs: (0) expression lacking in distal part of leg, (1) expression along length of leg*. **[R2011:395]**
580. Collier *expression in the intercalary segment: (0) absent, (1) present*.

Morphology

581. *Segmented body: (0) absent, (1) present*. **[M2009:19]**

582. *Two long tagmata expressed in the appendages (anterior batch of appendages longer than posterior batch): (0) absent, (1) present*. **[M2009:27]**
583. *Isolated sclerites/nodes: (0) absent, (1) present*. **[M2009:28]**
584. *More than two plates or nodes on each trunk segment: (0) absent, (1) present*. **[L2011:32]**
585. *Spine-shaped sclerites: (0) absent, (1) present*. **[M2009:29]**
586. *Papillae/setae on trunk annuli: (0) absent, (1) present*. **[M2009:32]**
587. *Number of annuli per segment: (0) few (up to 6-7 per segment), long, continuous around trunk, (1) many (9-25), short, weak or absent laterally.* **[R1998:6]**
588. *Segments progressively shorten posteriorly: (0) absent, (1) present*. **[R1998:8]**
589. *Prominent, unsclerotised nodes on trunk segments: (0) absent, (1) present*. A number of lobopodians possess enlarged, unsclerotized nodes on their dorsum. These nodes are typically associated with a pair of appendages. Nodes may either be paired, as in *Xenusion*, or in a tightly packed row of four, as in *Hadranax*.
590. *Antero- and posterodorsal spines on carapace: (0) absent, (1) present*. This character refers to the elongate carapace spines of *Isoxys.*
591. *Carapace divided into five distinct plates: (0) absent, (1) present*. The carapace of *Balanus*, *Semibalanus* and adult specimens of the Silurian *Rhamphoverritor* is divided into five distinct plates.
592. *Rostrum: (0) absent, (1) present*. **[R2001:10]**
593. *Anterior cephalic spines elongate: (0) absent, (1) present.* This character is only applicable to taxa with anterolateral cephalic spines (ch. 19), i.e. marrellids. Elongate anterior cephalic spines are present in *Mimetaster* and an undescribed marrellomorph from the Ordovician of Morocco.
594. *Cephalon composed of a small anterior plate and a posterior shield: (0) absent, (1) present*.
595. *Lateral cephalic furrow: (0) absent, (1) present*. The cephalon of branchiopods possesses a lateral furrow, giving the impression of possessing two distinct segments.
596. *Marginal rim ornamented: (0) absent, (1) present.* **[O2013:34]**
597. *Notches on margin of cephalic shield possibly indicative of original segmentation: (0) absent, (1) present*. The cephalon of mollisonids, particularly *Urokodia* and *Mollisonia symmetrica*, possesses distinct notches of spines along the lateral margins.
598. *Posterior cephalic band: (0) absent, (1) present*. **[O2013:37]**
599. *Cephalic furrows: (0) absent, (1) present*. **[O2013:38]**
600. *Vaulted cephalic shield: (0) absent, (1) present*. The cephalic shield of some aglaspidid arthropods is highly vaulted, i.e. anteroposteriorly short but dorsoventrally elongate.
601. *Eyes located close to medial margin of cephalic shield: (0) absent, (1) present.* The lateral eyes of the aglaspidids *Chlupacaris* and *Flobertia* are located on a central node on the cephalic shield. This is in contrast to other aglaspidids which typically have lateral eyes located towards the anterolateral margins of the cephalic shield.
602. *Fusion of prosoma and opisthosoma into a single functional unit: (0) absent,*

(1) present. In mites the prosoma and opisthosoma are fused into a single unit with little or no evidence of original segmentation.

603. *Number of thoracomeres involved in forming the cephalothorax: (0) no thoracomeres fused with the head, (1) one thoracomere fused with head and carapace (if present), (2) two thoracomeres fused with head and carapace: (3) three thoracomeres fused with head shield, (4) eight thoracomeres fused with head and carapace.* **[R2001:9]**
604. *Relative proportions of preabdominal tergites: (0) equal sized, (1) increase in area T1 to T4 (with T5 narrower), (2) increase in area T1 to T3 (with T4 and T5 progressively narrower), (3) tergites decrease in size posteriorly*. This character applies to euthycarcinoids only.
605. *Angle of anterolateral margin of preabdominal tergites (based on T2): (0) <100º, (1) >100º.* This character applies to euthycarcinoids only.
606. *Preabdominal ornamentation: (0) absent, (1) present*. This character applies to euthycarcinoids only.
607. *Visible monosomite: (0) absent, (1) present*.
608. *Reduced first tergite of trunk (microtergite): (0) absent, (1) present*. Both xiphosurans and eurypterids possess a reduced first tergite, the anterior part of which is underneath the posterior margin of the prosoma.
609. *Fusion of anterior trunk/opisthosomal tergites into a buckler: (0) absent, (1) present*.
610. *Buckler completely fused: (0) absent (evidence of original segmentation), (1) present*.
611. *Dorsal marginal trunk rim: (0) absent, (1) present*. This character describes a distinct margin on the fused dorsal shield of vachonisiids and skaniids. This character is present in all skaniids and vachonisiids but is absent from *Skania fragilis* (DAL pers. ob.).
612. *Trunk margins with dense setal fringe: (0) absent, (1) present*. A dense setal fringe is present on the trunk margin of the cheloniellid *Duslia* and an undescribed cheloniellid from the Ordovician of Morocco.
613. *Trunk covered by fine tuberculation: (0) absent, (1) present.* A finely tuberculate cuticle is possessed by a number of aglaspidids.
614. *Tuberculation on posterior margin of tergites: (0) absent, (1) present*. **[O2013:56]**
615. *Axial spines: (0) absent, (1) present*. **[O2013:57]**
616. *Thorax reduced or absent: (0) absent, (1) present*. This character is formulated to only be applicable to taxa with a pygidium.
617. *Thoracic keel: (0) absent, (1) present*.
618. *Elongate ventral spines on thoracic tergites: (0) absent, (1) present*. Both *Ecnomocaris* and *Thelxiopes* possess a row of elongate medial-ventral spines on their thoracic segments.
619. *Strengthening rays on lateral flaps: (0) absent, (1) present*. **[D2009:37]**
620. *Pleomere size: (0) first pleomere fully developed, of similar size and appearance of the more posterior pleomeres, (1) first pleomere reduced, smaller than the second pleomere, (2) more pleomeres reduced.* **[R2001:2]**

621. *Pleura of the second pleon segment: (0) pleura not overlapping other pleura, or pleura overlapping only those of the third pleomere, or pleura absent, (1) pleura of the second pleomere overlapping those of the first and third pleomere*. **[R2001:3]**
622. *Long axis of postabdominal somites: (0) width, (1) length, (2) width to length ratio changes extensively posteriorly.* This character refers only to the postabdomen of euthycarcinoids.
623. *Ornamented post-abdominal surface: (0) absent, (1) present*. This character applies to euthycarcinoids only.
624. *Extended posterior end (body axis projects posterior to ultimate pair of lobopods): (0) absent, (1) present*. **[M2009:23]**
625. *Spines on posterior margin of dorsal shield: (0) absent, (1) present*. This character is only applicable to taxa with a fused dorsal shield (ch. 69).
626. *Post-appendicular area bulbous and flap-like: (0) absent, (1) present*. This character refers to non-sclerotised panarthropods. The lobopodian *Jianshanopodia*, the dinocaridid *Pambdelurion*, and possibly also the lobopodian *Siberion*, possess a bulbous post-appendicular flap, differentiated from the main body axis by a narrow demarcation.
627. *Pygidium small, rounded and bordered by curved tergal pleura: (0) present, (1) absent*.
628. *Cephalon and pygidium isopygous and semi-circular: (0) absent, (1) present.*
629. *Segmentation of pygidium: (0) absent, (1) present*. The pygidia of *Soomaspis* and *Kangacaris* possess transverse furrows possibly indicative of original segmentation.
630. *Broad marginal rim on pygidium: (0) absent, (1) present*.
631. *Nature and number of lateral pygidial spines: (0) none (absent), (1) serrated margin, (1) two, (2) four, (3) four plus one medial, (4) three plus one medially.*
632. *Anterior expansion of tail spine: (0) absent, (1) present*. **[O2013:69]**
633. *Tail spine with medial cleft: (0) absent, (1) present*. **[O2013:72]**
634. *Paddle with projections: (0) absent, (1) present*. **[O2013:73]**
635. *Postventral plates: (0) absent, (1) present*. **[O2013:74]**
636. *Postventral plate medial attachment: (0) absent, (1) present*. **[O2013:82]**
637. *Lateral telson processes articulated: (0) absent, (1) present*.
638. *Furcal length: (0) elongate (extend beyond posterior trunk margin), (1) short (does not extend beyond posterior trunk margin).* This character only applies to taxa which possess dorsal pre-telson furcae, i.e. cheloniellids.
639. *Articulated cerci with elongate setae: (0) absent, (1) present*.
640. *Differentiated uniramous frontal appendage: (0) absent, (1) present*. In onychophorans, dinocaridids, arthropods, and some onychophorans the anteriormost appendages differ in morphology from the trunk appendages and from other cephalic appendages.
641. *Grasping limbs: (0) absent, (1) present*. **[M2009:4]**
642. *Spinose great appendage style: (0) unpaired annulated proboscis-like structure with branching tip, (1) around 15 segments with a double row of short spines, (2) single row of elongate ventral spines with fewer than 15 articles, (3) annulated*

lobopodous, (4) reduced number of subpentagonal podomeres. This character was modified to include states observed in *Isoxys*,
namely state 4 which is present in *I. acutangulus* and *I. curvirostratus*. **[***cf.* **D2009:23]**

643. *Relative orientation of appendicular ("great-appendage") spines compared to appendage podomere: (0) perpendicular, (1) chelate or sub-chelate*.
644. *Orientation of appendicular spines relative to the body: (0) ventral, (1) dorsal*.
645. *Elongate basal spine on great-appendage: (0) absent, (1) present*.
646. *Great-appendage geniculate: (0) absent, (1) present*.
647. *Number of segments in "short-great-appendage": (0) two promimal elements plus four spinose distal elements, (1) two plus three, (2) two plus two, (3) two plus one, (4) five or more with an undifferentiated peduncle*.
648. *Endopods multiannulated with elongate setae: (0) absent, (1) present*. This character refers to the setiferous endopods of euthycarcinoids.
649. *Cephalic exopods longer than endopods: (0) absent, (1) present*.
650. *Preabdominal appendage length: (0) equal, (1) shortening posteriorly, (2) lengthening posteriorly.*
651. *Elongate exopod tipped with setal "brush": (0) absent, (1) present.* The elongate exopods of some marrellomorph arthropods, particularly skaniids and vachonisiids, are tipped with a concentration of filamentous setae, often attached to a broad semi-lunate base, reminiscent of a sweeping brush.
652. *Nature of second (first post-antennal/raptorial) cephalic appendages: (0) biramous walking leg or antennae, (1) uniramous walking legs.* **[O2013:6]**
653. *First post-antennal limbs with proximal endite in early larvae: (0) absent, (1) present*. **[H2010:6]**
654. *First post-antennal limbs with proximal endite in later developmental stages: (0) absent, (1) present*. **[H2010:7]**
655. *Proximal endite enlarged to form coxa in first post-antennal limb: (0) absent, (1) present*. **[H2010:8]**
656. *First post-antennal exopod with 3-2 setation pattern: (0) absent, (1) present*. **[H2010:10]**
657. *Nature of third (second post-antennal/raptorial) cephalic appendages: (0) biramous walking legs, (1) uniramous walking legs.* **[O2013:7]**
658. *Second post-antennal limb with proximal endite in early larvae: (0) absent, (1) present*. **[H2010:13]**
659. *Third post-antennal limb with proximal endite in early larvae: (0) absent, (1) present*. **[H2010:20]**
660. *Third post-antennal limb with proximal endite in later developmental stage: (0) absent, (1) present*. **[H2010:21]**
661. *Third post-antennal limb with setiferous multi-annulated exopod: (0) absent, (1) present*. **[H2010:22]**
662. *Fourth post-antennal limb with proximal endite in early larvae: (0) absent, (1) present*. **[H2010:26]**
663. *Modified cephalic appendages with micro-hooks for host attachment: (0) absent, (1) present*.

664. *Antennular exopod morphology: (0) up to six annuli each bearing a seta, (1) two podomeres, (2) one plus two podomeres, (3) with a scale bearing setae, (4) spinneret seta.*
665. *Ratio between the tarsus and the tibia of the opilionid palp: (0) subequal, (1) tarsus shorter than tibia, (2) tarsus longer than tibia*. **[G2002:61]**
666. *Mandible with row of teeth along inner edge: (0) absent, (1) present*. **[O2009:15]**
667. *Segmentation of endopod on mandible: (0) six segments, (1) five segments*.
668. *Lacinia mobilis on larval (embryonic) mandible: (0) absent, (1) present*. **[R2001:15]**
669. *Toothed endite on mandible: (0) absent, (1) present*.
670. *Maxillula with 1+3 endites: (0) absent, (1) present*.
671. *Second maxilla exopod: (0) present, forming a broad plate, (1) modified to scaphognathite, (2) absent*. **[R2001:18]**
672. *Second maxilla palp: (0) present, (1) vestigial or absent*. **[R2001:19]**
673. *Maxillary exite: (0) segmented, (1) brachial plate, (2) single segment*.
674. *Exopod on maxilliped: (0) absent, (1) present*.
675. *First thoracopod: (0) unmodified appendage, (1) transformed into a maxilliped*. **[R2001:21]**
676. *Additional thoracopods transformed into maxillipeds: (0) absent, (1) second (and third) thoracopod modified into maxillipeds, (2) five 'maxillipeds'.* **[R2001:22]**
677. *Locomotory limbs with spinules/papillae: (0) absent, (1) present*. **[M2009:9]**
678. *Terminal claws on appendages: (0) absent, (1) present*. **[M2009:10]**
679. *More than two claws per limb: (0) absent, (1) present*. **[M2009:11]**
680. *One pair of limbs behind the last pair/set of sclerites: (0) absent, (1) present*. **[M2009:30]**
681. *Trunk limbs with appendiculae or branches: (0) absent, (1) present*. **[L2011:18]**
682. *Reduced sixth appendage: (0) absent, (1) present*.
683. *Epipodite on sixth appendage: (0) absent, (1) present*.
684. *Phyllopodous appendages: (0) absent, (1) present*.
685. *Number of pleon appendages: (0) six, (1) three (third to fifth pleopods missing)*. **[R2001:4]**
686. *First thoracopod exopod: (0) present, (1) absent*. **[R2001:23]**
687. *First thoracopod epipodite: (0) epipodite respiratory and differs in general nit from the epipodites of more posterior thoracopods, (1) epipodite produces a respiratory current, it is respiratory itself or non-respiratory, (2) epipodite present, but not producing a respiratory current and non-respiratory, (3) epipodite absent*. **[R2001:24]**
688. *Thoracopods 2-8, number of epipodites (without oostegites) on at least some thoracopods: (0) one, (1) two (often two branches, developing from one anlage) present, (2) more than two epipodites, typically an arthrobranchiae and pleurobrachiae, (3) none.* **[R2001:25]**

689. *Thoracopods 2-8, position of epipodites: (0) lateral, (1) at least one branch carried under the thorax.* **[R2001:26]**
690. *Exopod on thoracopods 2-8: (0) present, at least on some thoracopods, flagellate or vestigial, (1) completely absent from all thoracopods.* **[R2001:30]**
691. *Thorax-coxa articulation: (0) allows promotion/remotion, (1) allows abduction/adduction*. **[R2001:31]**
692. *Coxa-basis articulation: (0) dicondylic, allows abduction/adduction, (1) monocondylic, motion in different direction is possible*. **[R2001:32]**
693. *Intrabasal articulation: (0) absent, (1) present*. **[R2001:33]**
694. *Coxal plates on thoracopods: (0) absent, (1) present*. **[R2001:34]**
695. *Thoracopods 4-5 chelate: (0) absent, (1) present*. **[R2001:35]**
696. *Thoracopod 6 chelate: (0) absent, (1) present*. **[R2001:36]**
697. *Pleopods: (0) biramous, (1) uniramous*. **[R2001:37]**
698. *Appendicules internae on the endopods: (0) absent, (1) present*. **[R2001:38]**
699. *Vermiform cleaning thoracopod: (0) absent, (1) present*.
700. *Multi-faceted eyes: (0) absent, (1) present*. This character is not the same as ch. 354, which refers to compound eyes only. The present character also refers to the clusters of ocelli observed in some lobopodian taxa[46].
701. *Single enlarged lateral eye: (0) absent, (1) present*.
702. *Number of globuli cell clusters in the deutocerebrum associated with the offactory lobe: (0) one, (1) two.* **[R2001:44]**
703. *Number of optic neuropils: (0) two, (1) three*. **[R2001:47]**
704. *Ultrastructure of ommatidia: (0) bipartite cone completely round in transverse sections, cone without any extensions, (1) cone with two lateral extensions (in transverse section button-like), formed by one cone cell each.* **[R2001:49]**
705. *Nauplius eye* sensu stricto*: (0) absent, (1) present*. **[R2001:53]**
706. *Bellonci organ: (0) absent, (1) present*.
707. *Ventral frontal organ: (0) present, (1) absent*. **[R2001:55]**
708. *Antennal gland: (0) present, (1) absent.* **[R2001:57]**
709. *Maxillary gland: (0) present, (1) absent*. **[R2001:58]**
710. *Oral papillae: (0) absent, (1) present*. **[L2011:2]**
711. *Oral cone: (0) absent, (1) present*.
712. *Structure of mouth part apparatus (ch. 405): (0) variable number of undifferentiated plates, (1) 32 plates with differentiation of four enlarged plates in a cross arrangement.* Investigation of the oral circlet of *Anomalocaris* showed it should be coded as state 0. **[D2009:8]**
713. *Specialised cephalo-thoracic feeding apparatus: (0) absent, (1) present*. Copepods, mystacocarids and the Cambrian “Orsten” taxon *Skara* possess a similar feeding system.
714. *Labral posterior tooth: (0) absent, (1) present*. **[R2001:16]**
715. *Gnathosoma: (0) absent, (1) present*. **[S2007:32]**
716. *Formation of the midgut: (0) by entoderm, (1) at the border between stomodaeum and proctodaeum*. **[R2001:73]**
717. *Pleon musculature: (0) simple, (1) precaridoid, (2) cardioid*. **[R2001:59]**

718. *Number of pairs of ostia: (0) more than five, (1) five, (2) three, (3) two, (4) one, (5) zero*. **[R2001:61]**
719. *Extension of heart: (0) in whole thorax (head in part) and pleon, (1) thorax, (2) only posterior part of thorax and pleon*. **[R2001:62]**
720. *General form of heart: (0) long, extended, (1) bulbous, (2) simple, short.* **[R2001:63]**
721. *Arteria subneuralis/supraneuralis: (0) absent, (1) present*. **[R2001:64]**
722. *Aorta descendens: (0) undivided sternal artery passes through the nervous system, (1) sternal artery branches off into three branches dorsal of the ventral nervous system, all branches pass separately through the nerve cord*. **[R2001:66]**
723. *Atrium between the inferomediana connecting the cardac primary filter grooves with the pyloric filter grooves: (0) absent, (1) present*. **[R2001:71]**
724. *Number of secondary filter grooves in the inferomedianum posterius: (0) numerous, (1) eight to six, (2) three, (3) two, (4) one.* **[R2001:72]**
725. *Carapace: (0) non-respiratory, (1) with respiratory function*. **[R2001:7]**
726. *Respiratory function in pleopods: (0) absent, (1) present*. **[R2001:41]**
727. *Cylindroleberidid-type gills: (0) absent, (1) present*.
728. *Position of repugnatorial glands: (0) on the side of the cephalic shield, visible from above, (1) below the side of the cephalic shield, not visible from above*. **[G2002:13]**
729. *Subdivision of ovary into a well-defined anterior germarium and an elongate vitellarium: (0) absent, (1) present.*
730. *Localization of the germaria within the trunk and/or head region of the body: (0) absent, (1) present*.
731. *Unpolarized somatic cells accommodating germline cells: (0) absent, (1) present*.
732. *Ventral brood pouch: (0) absent, (1) present*. **[O2009:56]**
733. *Oostegite position: (0) on thoracopods 2-8, (1) on thoracopods 2-6, (2) on thoracopods 3-6.* **[R2001:28]**
734. *Reduction of the oostegites after every brood: (0) absent, (1) present*. **[R2001:29]**
735. *First and/or second pleopods modified for sperm transfer in males: (0) absent, (1) somatopod petasma, including modification of the exopods of the second pleopod, (2) endopod of the first pleopod completely modified for sperm transfer, modification different in the second endopod*. **[R2001:39]**
736. *Sperm nuclear membrane: (0) present, (1) absent, chromatine diffuse.* **[R2001:77]**
737. *Sperm microtubular arms or spikes: (0) absent, (1) present*. **[R2001:78]**
738. *Cross striated perforatorium (possible centriolar root homologous): (0) absent, (1) present*. **[R2001:79]**
739. *Spermatophore: (0) absent, (1) present*. **[R2001:80]**
740. *Ovipositor morphology: (0) segmented (jointed type), (1) unsegmented (unjointed type)*. **[G2002:173]**

Development

741. *Direct development: (0) absent, (1) present*. **[R2001:82]**
742. *Free-living nauplius larva: (0) present, (1) absent*. **[R2001:83]**
743. *Development of appendages: (0) advanced development of anterior head appendages, (1) continuous anteroposterior decrease in the degree of appendage formation*. **[R2001:84]**
744. *Number of ectoteloblasts: (0) 19, (1) variable, (2) none*. **[R2001:86]**
745. *Arrangement of ectoblasts: (0) forming a ring around the caudal papilla (giving rise to embryonic ventral and dorsal material), (1) forming a transverse row (only the ventral side of the embryo is formed by ectoteloblsts and the dorsal side is closed much later in development)*. **[R2001:87]**
746. *Folding of early embryo (nauplius larva): (0) ventrally folded, (1) dorsally folded*. **[R2001:88]**
747. *Yolk distribution in the embryo: (0) posterior part of the embryo contains no yolk, (1) posterior part of the embryo contains yolk*. **[R2001:89]**
748. *Number of thoracic appendages in hatchling: (0) eight, (1) seven, (2) six*. **[2001:90]**
749. *Embryonic dorsal organ: (0) present, (1) absent*. **[R2001:91]**
750. *Form of embyonic dorsal organ: (0) simple layer, (1) cup-shaped*. **[R2001:92]**
751. *Transient paired dorsal organs: (0) absent, (1) present*. **[R2001:93]**
752. *Cyprid larva: (0) absent, (1) present*.

Behaviour

753. *Brood care: (0) absent, (1) brood care with thoracopods without feeding by the mother, (2) brood care attaching the eggs to the pleopods, (3) brood care using a dorsal brood pouch, (4) brood care using a marsupium formed by oostegites, (5) brood care using elongated first pleopod.* **[R2001:81]**

From Briggs et al. (2016)

754. *Posterior transverse ridge on tergites: (0) absent, (1) present*.
